# Directional Confinement Governed Ion Transport for Efficient Actuation

**DOI:** 10.1002/adma.75384

**Published:** 2026-10-15

**Authors:** Yufei Zhang, Qiuchun Lu, Adit Gupta, Sen Wang, Xueyang Wang, Jian Yu, Xuanyi Hu, Daniel Mandler, Pooi See Lee

**Affiliations:** ^1^ School of Materials Science and Engineering Nanyang Technological University Singapore Singapore; ^2^ Singapore‐HUJ Alliance for Research and Enterprise (SHARE) Smart Grippers for Soft Robotics (SGSR) Campus for Research Excellence and Technological Enterprise Singapore Singapore; ^3^ Institute of Chemistry The Hebrew University of Jerusalem Jerusalem Israel

**Keywords:** directional confinement, electrochemical deformation, ion‐transport networks, porous electrolyte, structural anisotropy

## Abstract

Ionic transport kinetics in electrolytes are a key determinant of ionic actuator performance. Conventional random porous electrolytes are constrained by porosity and tortuosity, leading to an intrinsic limitation in ion mobility within the Bruggeman framework. Here, structural anisotropy is introduced to establish a unified framework that analyzes ion transport from random diffusion to directionally confined regimes. To validate this concept, anisotropic electrolytes with oriented channels are fabricated via directional freeze‐drying. These structures exhibit pronounced directional dependence, with through‐plane ion mobility twice higher than that of the disordered counterparts, leading to significantly enhanced ionic actuator performance and mitigating the inherent trade‐off between strain and blocking force. Moreover, the directional confinement effect is observed across multiple polymer systems, highlighting the critical role of structural anisotropy in regulating ion transport and offering a framework for enhancing electrolyte performance through microstructural design. This strategy enables environmentally robust and stable operation of soft electrochemical actuators for grippers and biomimetic robotics.

## Graphical abstract

Structural anisotropy provides an effective means to regulate ion transport in polymer electrolytes. Directionally aligned channels formed by freeze‐drying provide continuous pathways for ion migration, enhancing ion mobility and simultaneously improving the strain and blocking force of ionic actuators. This structure‐guided strategy offers a general approach to high‐performance electrolytes for soft electrochemical actuators.

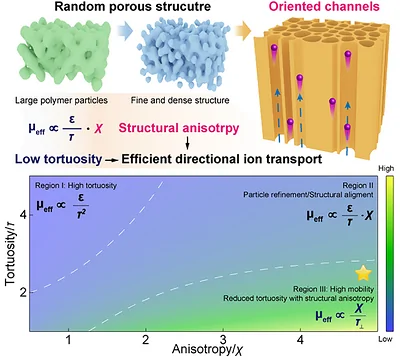

## 1 Introduction

Soft actuators, often referred to as artificial muscles, are fundamental components of soft robotics, enabling systems to perform delicate, adaptive, and biologically inspired movements [1, 2, 3, 4, 5, 6, 7]. They can be broadly classified into electronic and ionic types based on their actuation mechanisms. Electronic actuators, including electrostatic [8, 9, 10], piezoelectric [11, 12], or thermal [13, 14, 15, 16, 17] systems, show fast response and high energy output, but are limited by high driving voltage, structural rigidity, or restricted deformation. In contrast, ionic actuators operate at low voltage (<2 V), provide large bending deformation and inherent compatibility with sensing, making them highly attractive for flexible electronics [18, 19, 20, 21, 22, 23, 24, 25, 26]. An ionic actuator consists of a sandwich structure comprising two electrodes and an intermediate electrolyte layer. Upon the application of voltage, mobile ions within the electrolyte migrate toward the counter electrode, inducing asymmetric swelling and bending [27, 28, 29]. Its actuation performance is jointly governed by the electrical conductivity and charge storage capacity of the electrodes, the size and mobility of ions, ion transport within the electrolyte, and interfacial adhesion between different layers. Accordingly, much effort has been devoted to optimizing the electrode, ranging from noble metals [30, 31, 32, 33, 34, 35] and conducting polymers [36, 37, 38, 39, 40] to carbon‐based nanocomposites [41, 42, 43], to improve electrical conductivity and capacitance. However, because actuation is mediated by ion transport and redistribution, the electrolyte composition and transport architecture are equally important. Ionic actuators can be broadly classified into aqueous and ionic‐liquid‐based systems [44]. Aqueous electrolytes offer high ionic conductivity and rapid actuation but suffer from evaporation, hydration dependence, and a narrow electrochemical stability window. Ionic liquids instead provide negligible vapor pressure and broad electrochemical stability windows, enabling durable operation in air [45, 46]. Importantly, their viscosity, ionic conductivity, and ion structure regulate ion migration and interfacial accumulation, thereby determining the response speed and deformation of the actuator. Among them, 1‐ethyl‐3‐methylimidazolium tetrafluoroborate (EMIM: BF_4_) is widely used because its relatively small and mobile ions provide a favorable balance between ionic conductivity, viscosity, and electrochemical stability [47]. Nevertheless, incorporating a conductive ionic liquid into a polymer matrix does not necessarily ensure rapid ion transport, which can remain restricted by polymer–ion interactions and tortuous transport pathways. For example, increasing electrolyte thickness generally extends the ion transport, reducing response speed or strain, although the accompanying increase in structural stiffness may enhance the blocking force. Therefore, regulating the electrolyte structure to promote through‐plane ion transport while maintaining mechanical integrity remains important for improving response speed, deformation, and force output in high‐performance ionic actuators.

To date, polymer electrolytes for ionic actuators have relied mainly on ionomers, among which Nafion is one of the most extensively studied due to its unique sulfonic acid‐based ion transport channels, excellent mechanical properties, and outstanding chemical stability [48, 49]. Doping and structure modification have therefore been explored to improve its ionic conductivity and actuation efficiency [50, 51, 52]. For instance, Nguyen et al. developed a functionally antagonistic polyelectrolyte based on Nafion and proper solvent, which showed continuous ion‐conducting networks, achieving a 36‐fold increase in bending displacement and a 10‐fold enhancement in response speed compared to conventional casting Nafion films [51]. Lian et al. incorporated high aspect ratio graphene oxide (GO) nanosheets into Nafion matrix, resulting in a GO/Nafion actuator with a blocking force four times higher than that of pristine Nafion [53]. However, ion transport in Nafion relies strongly on water molecules within its hydration shells. This fundamental dependence renders Nafion‐based actuators acutely sensitive to environmental humidity and particularly vulnerable to performance degradation at elevated temperatures due to the collapse of hydrated transport channels. To tackle these challenges, considerable efforts have been devoted to developing novel electrolyte materials. Recent studies have explored covalent–organic frameworks (COFs) [54, 55] and polymer network structures [56, 57, 58], including interpenetrating polymer networks (IPNs) and semi‐IPNs, to improve ionic conductivity and mechanical robustness. Yu et al. reported a sulfonic acid‐functionalized covalent organic framework with highly ordered 3.4 Å hexagonal channels, enabling efficient ion transport and rapid actuation under ± 0.5 V [59]. Although the above advanced polymer electrolytes demonstrate impressive actuation performance, the associated synthetic routes are often complex and involve nanoscale structural engineering, and their performance would decrease when applied to thicker electrolytes.

These limitations have stimulated the development of non‐aqueous ion‐gel electrolytes, in which ionic liquids are immobilized within polymer hosts such as poly(vinylidene fluoride‐co‐hexafluoropropylene) (PVDF‐HFP) [60, 61]. The HFP units disrupt regular polymer‐chain packing and increase the amorphous regions available for ionic‐liquid accommodation. In imidazolium‐based systems, reversible interactions between the cations and C–F dipoles further improve compatibility and provide transient physical crosslinks [62]. Consequently, PVDF‐HFP/ionic‐liquid electrolytes have been implemented as bucky‐gel [47], phase‐separated porous membranes [63], and electrospun fibrous networks [64]. However, their actuation dynamics depend not only on the ionic liquid content but also on the orientation and tortuosity of the ion‐transport pathways. Studies of ion‐conducting membranes indicate that through‐plane aligned channels can provide shorter and more continuous transport pathways than isotropic structures [65]. Ice templating offers a practical route to such architectures, whereby directional freezing guides solvent‐crystal growth and subsequent sublimation preserves the resulting aligned pores [66]. Ion transport in porous electrolytes is often described using classical effective‐medium models developed for disordered or effectively isotropic microstructures. Within these descriptions, effective ion mobility is governed primarily by porosity and tortuosity, as represented by Bruggeman‐type relations [67, 68]. Although useful for conventional porous networks, these scalar parameters do not explicitly represent the direction‐dependent transport arising from structural anisotropy. Incorporating anisotropy into conventional porosity–tortuosity descriptions may therefore provide a more appropriate framework for understanding directionally confined ion transport and its relationship with actuator performance.

Here, an anisotropy factor is incorporated into the classical ion transport framework to provide a unified description of ion migration spanning from random diffusion to directionally confined transport. Theoretical analysis indicates that oriented channels can substantially enhance through‐plane ion mobility, exceeding the improvement achievable by tortuosity reduction alone. To validate this concept, anisotropic porous PVDF‐HFP electrolytes are fabricated via directional freeze‐drying, revealing ion mobility along the channels more than twice that in disordered structures. This enhancement is further observed in other polymer systems (TPU and PVDF), indicating the generalizability of the proposed strategy. Ionic actuators based on the oriented‐channel electrolytes exhibit concurrent improvements in strain and blocking force, thereby mitigating the conventional trade‐off between response speed and mechanical output. Benefiting from the hydration‐independent transport mechanism, the actuator shows enhanced thermal stability and maintains stable performance where Nafion‐based actuators fail. Systematic optimization identifies an optimal ratio (9 wt% PVDF‐HFP, 1.5 ∼ 2.0 ionic liquid ratio) that maximizes the structural advantages and enhances actuation performance. The practical utility of these actuators is demonstrated through their integration into soft grippers, steerable guidewires, and untethered biomimetic robotic fish. Grippers fabricated from anisotropic electrolytes exhibit enhanced gripping capability, enabling the manipulation of objects that cannot be handled by casting film‐based devices, while maintaining stable performance at elevated temperatures. In addition, the robotic fish driven by these actuators achieves a swimming speed of 0.07 body lengths per second, delivering efficient propulsion at a low actuator‐to‐body mass ratio (∼0.5%). These results indicate that the construction of oriented channels facilitates rapid ion transport and enhances actuator performance. Overall, a direct correlation between microstructural anisotropy and ion transport behavior is established, suggesting that directional confinement enabled by structural design rather than complex chemical modification could effectively enhance actuator performance, thereby providing a broad strategy for designing high‐performance electrolytes in soft electrochemical systems.

## 2 Results

### 2.1 Evolution of Ion Transport Mechanisms in Porous Electrolytes

Ion transport kinetics in electrolytes fundamentally govern the performance of electrochemical and soft actuation systems. Figure 1a illustrates two representative architectures: random porous electrolytes and oriented channel electrolytes. For the former, within the Bruggeman framework, ion mobility is constrained by porosity (*ε*) and tortuosity (*τ*). Although densification of the microstructure can reduce tortuosity and moderately enhance transport, the resulting improvement remains limited. This limitation has motivated the development of electrolytes with oriented channels to enable faster ion transport. However, the above theories based on isotropic assumptions fail to accurately describe ion transport in oriented channel systems. To establish a unified framework describing ion transport across random diffusion and directionally constrained regimes, an anisotropy factor (*χ*) is introduced into the model. This framework predicts that anisotropic films with oriented channels can markedly enhance through‐plane ion transport in ionic actuators, thereby improving device performance (Figure 1b). To validate this hypothesis, ion transport in random and anisotropic structures is comparatively analyzed along both through‐plane and in‐plane directions. Ions are initially placed on one side, and an external voltage is applied across the electrolyte. The results show that both structures exhibit disordered characteristics in the in‐plane direction, leading to comparable mobility rates. In contrast, along the through‐plane direction, the oriented channels in anisotropic structure enable significantly faster ion transport (Figure 1c). Quantitative tracking of through‐plane ion concentration across different models further confirms that the oriented channel structure exhibits the most rapid concentration variation, attributable to reduced transport resistance associated with low‐tortuosity pathways (Figure 1d). Further simulation details on ion transport can be found in Sections SA and SB and Movie S1 of Supporting Information.

**FIGURE 1:**
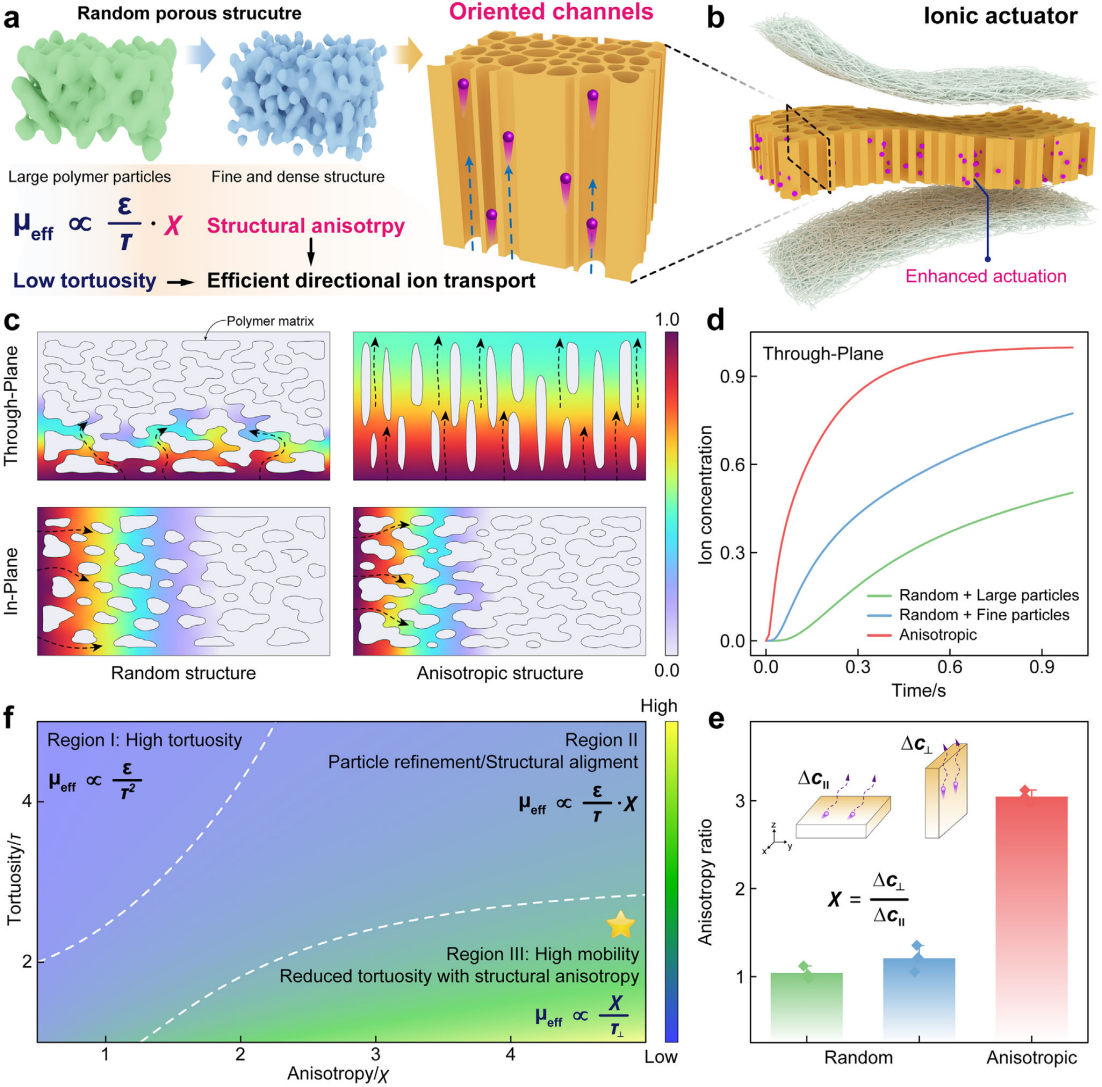
Mechanistic evolution of ion transport in porous electrolyte. (a) Structural evolution of porous microstructures from random distributions to oriented channels. Ion mobility in random porous systems is primarily governed by porosity (*ε*) and tortuosity (*τ*), with larger domains leading to higher tortuosity and thus hindered ion transport. For aligned structures, an anisotropy factor (*χ*) is introduced to more accurately describe the preferential, directionally guided ion transport. (b) Schematic illustration of an ionic actuator based on the oriented porous electrolyte, highlighting enhanced through‐plane ion transport and improved actuation performance. (c) Simulated ion concentration distributions comparing random and anisotropic structures under through‐plane and in‐plane transport conditions. The anisotropic structure exhibits spatially confined and continuous ion transport pathways, whereas random structures show dispersed and diffusion‐limited transport behavior. This distinction is further corroborated by quantitative comparison of concentration evolution along the through‐plane direction across the different models (d). (e) Quantification of structural anisotropy based on the ratio of through‐plane to in‐plane ion concentration variations. Random structures exhibit an anisotropy factor of ∼ 1, whereas oriented channels reach values of ∼ 3, indicating enhanced directional ion transport. (f) Transport regime map describing the interplay between tortuosity and anisotropy. Three regimes are identified: (i) a high tortuosity regime dominated by diffusion with pronounced tortuosity penalties; (ii) an intermediate regime where reduced particle size or emerging alignment enhances ion mobility; (iii) a high anisotropy regime in which fast ion transport along oriented channels becomes dominant.

The anisotropy factor (*χ*) is defined as the ratio of through‐plane to in‐plane ion concentration variation rates (Figure 1e). For random disordered structures, *χ* remains close to 1 regardless of particle size, indicating nearly isotropic ion transport. In contrast, the anisotropic structure exhibits a value of ∼ 3, demonstrating a pronounced directional preference in ion transport. Based on these analysis, a transport regime map is constructed to elucidate the interplay between tortuosity and anisotropy (Figure 1f), delineating three distinct regimes: (i) Tortuosity‐dominated regime, corresponding to random disordered structures with high *τ*, where ion mobility is strongly limited by tortuosity; (ii) Transition regime, where reduced particle size or emerging alignment lead to ion mobility governed by the combined effects of *τ* and *χ*; (iii) Directional confinement regime, corresponding to oriented channels, where low *τ* together with high *χ* significantly enhances ion transport along the channel direction.

### 2.2 Structure–Property Relationship in PVDF‐HFP Electrolytes

PVDF‐HFP was selected as the matrix material owing to its good chemical stability, processability, and solvent compatibility. By controlling the solvent system and fabrication process, PVDF‐HFP membranes with distinct morphologies can be constructed, providing an experimental basis for systematically investigating the relationship between membrane structure and ion transport. In this study, DMAc (*N,N*‐dimethylacetamide) and 1,4‐dioxane are used as solvents to prepare casting and directional freeze‐drying membranes with different structures. Detailed physical properties of these two solvents are listed in Table S1, and the fabrication process of the directional freeze‐drying membrane is illustrated in Figure S4. For clarity and ease of comparison, the three PVDF‐HFP membranes were designated as DMAc‐cast, Dioxane‐cast, and Dioxane‐FD according to the casting solvent and membrane fabrication method. As shown in Figure 2a,b, the DMAc‐cast membrane exhibits large granular domains with a relatively rough and slightly opaque appearance, whereas the Dioxane‐cast membrane shows a denser microstructure and higher optical transparency. Notably, the relatively high freezing point of 1,4‐dioxane (11.8°C) enables directional freeze‐drying, yielding a white, opaque film arising from the formation of the internally oriented porous structure. The membranes exhibit similar XRD patterns and TGA profiles (Figures S5 and S6), indicating no significant differences in their predominant crystal structures and thermal degradation stability. In contrast, their crystallization behaviors differ markedly. The DMAc‐cast membrane exhibits a broad crystallization peak at approximately 70°C–75°C, indicating that crystallization requires greater supercooling and occurs over a broader temperature range. Combined with the SEM observations, the large domains are more likely associated with greater compositional rearrangement and morphological coarsening during the slow removal of DMAc. Cross‐sectional SAXS data provides more structural insights (Figure 2c). While the casting membranes (DMAc‐cast and Dioxane‐cast) show circular scattering patterns of isotropic structures, the freeze‐dried (Dioxane‐FD) membrane exhibits an elliptical scattering pattern, indicating the structural anisotropy along the freezing direction. N_2_ adsorption isotherms and porosity data in Figure 2d confirm the porous structure of the membranes. The Dioxane‐FD film exhibits a specific surface area of 2.43 m^2^/g, nearly ten times that of the conventionally casting membranes, along with a porosity of ∼ 70.5%, providing abundant space for ion storage. The mechanical properties (Figure 2e) show that the dense membrane prepared by Dioxane‐cast exhibits the highest Young's modulus and strain, whereas the Dioxane‐FD membrane shows a reduced modulus and stretchability due to its porous structure. Detailed mechanical properties and Fourier transform infrared spectroscopy of the three PVDF‐HFP electrolytes at different concentrations are provided in Figures S7 and S8. Figure 2f presents the through‐plane ion mobility estimated from the current decay rate. The DMAc‐cast electrolyte exhibits the lowest ion mobility of 9.19 × 10^−6^ cm^2^ V^−1^ s^−1^. As the granular domains are reduced in the Dioxane‐cast system, the ion mobility increases to 1.38 × 10^−5^ cm^2^ V^−1^ s^−1^. The Dioxane‐FD membrane shows a further increase to 2.20 × 10^−5^ cm^2^ V^−1^ s^−1^, more than twice that of the DMAc‐cast sample and markedly higher than its in‐plane value (Figure S9). The combination of higher ion mobility and increased ionic liquid uptake arising from the porous structure results in the highest ionic conductivity in the freeze‐dried film. Importantly, compared to the casting films, the Dioxane‐FD samples exhibit markedly higher anisotropy ratios in both ionic conductivity and mobility (Figure 2g; Figure S10), with an anisotropy factor of approximately 2.6, close to the simulated value of ∼ 3. This agreement indicates that structural anisotropy effectively enhances ion transport along the oriented channels. Similar trends are observed across different polymer systems (TPU and PVDF), indicating the generalizability of this approach. Overall, these results validate the proposed structure–transport relationship, confirming that directionally confined channels effectively enhance ion transport.

**FIGURE 2:**
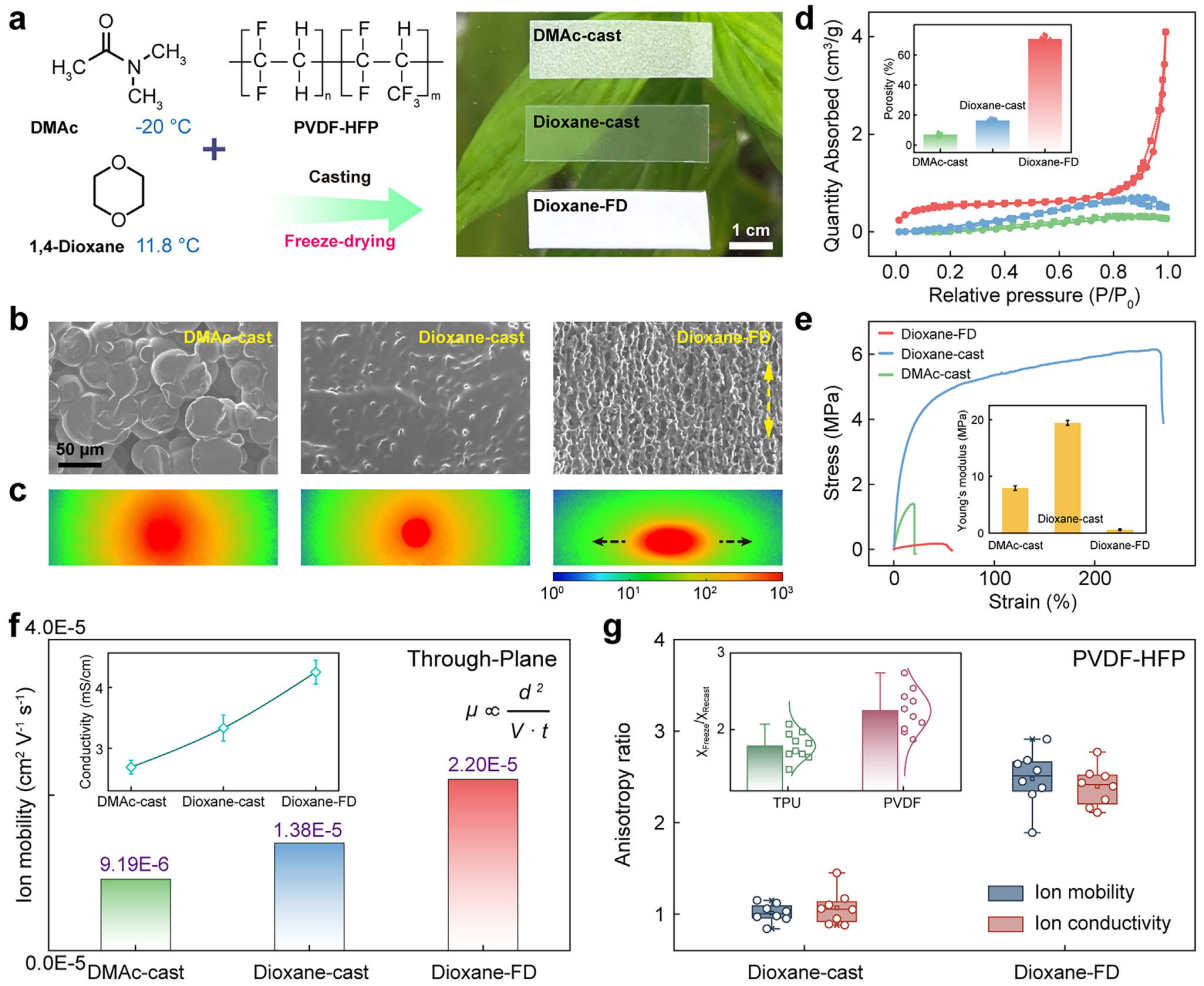
Characterization of electrolytes with different morphologies. (a) Optical images of PVDF‐HFP membranes fabricated by DMAc‐cast, Dioxane‐cast, and Dioxane‐FD, with the corresponding cross‐sectional SEM images shown in (b). Distinct microstructures arise from the different processing strategies: DMAc‐cast membranes exhibit pronounced granular domains with a slightly opaque appearance, whereas Dioxane‐cast membranes display a dense morphology with higher transparency. The Dioxane‐FD membrane appears opaque with a clearly oriented porous structure. (c) Two‐dimensional SAXS patterns show that the casting films exhibit isotropic scattering, whereas only the Dioxane‐FD membrane displays an elliptical pattern, further confirming its anisotropic microstructure. (d) Nitrogen adsorption–desorption isotherms of PVDF‐HFP electrolytes, with the inset comparing porosity, confirm that the Dioxane‐FD film possesses a highly porous structure with an increased specific area. (e) Tensile stress‐strain curves and corresponding Young's modulus of PVDF‐HFP electrolytes, showing that the porous structure reduces stiffness and deformation capability. (f) Ion mobility estimated from current decay reveals that the freeze‐dried membrane exhibits more than twice that of the DMAc‐cast membrane, emphasizing the importance of directional ion transport pathways; the inset further shows that the Dioxane‐FD membrane achieves the highest ionic conductivity. (g) Anisotropy ratios of through‐plane and in‐plane ionic conductivity and mobility in PVDF‐HFP electrolytes, demonstrating that oriented channels induced by freeze‐drying confer significant advantages in ion transport; extended analysis across additional materials (TPU and PVDF) further confirms the generality of anisotropy in enhancing directional ion transport.

### 2.3 Actuator Performance Based on Different Electrolytes

The ionic actuator is fabricated by hot‐pressing the electrolyte and electrodes to validate the benefits of the directionally confined channels. The specific preparation procedure is described in the Method section and Figures S11 and S12. The experimental setup for displacement measurements is illustrated in Figure 3a, where the bending deformation is monitored using a laser displacement detection system. Before the measurements, cyclic voltammetry (CV) and electrochemical impedance spectroscopy (EIS) of different actuators are analyzed (Figure 3b,c). The Dioxane‐FD PVDF‐HFP electrolytes exhibit the largest enclosed area and lowest impedance among the samples, indicating that the oriented porous structure enhances the ion accumulation and charge storage capability while facilitating ion transport within the electrolyte. For a more systematic evaluation of device performance, strain is adopted as a normalized metric to analyze the actuation responses under different frequencies (0.05–10 Hz) and voltages (0.5–3 V), as shown in Figure 3d,e. The results clearly indicate that the ionic actuator with freeze‐dried film outperforms the other two devices, achieving ∼1.06% strain at 2 V and 0.05 Hz. Although higher voltages yield larger deformation, the ionic liquid used in this study (EMIM: BF_4_) is susceptible to degradation at elevated potentials. Therefore, the voltage is limited to 2 V to ensure stable performance, at which the blocking force is measured (Figure 3f). The Dioxane‐FD membrane actuator generates a blocking force of ∼1.2 mN, about three times that of the DMAc‐cast device, providing sufficient force for practical object manipulation.

**FIGURE 3:**
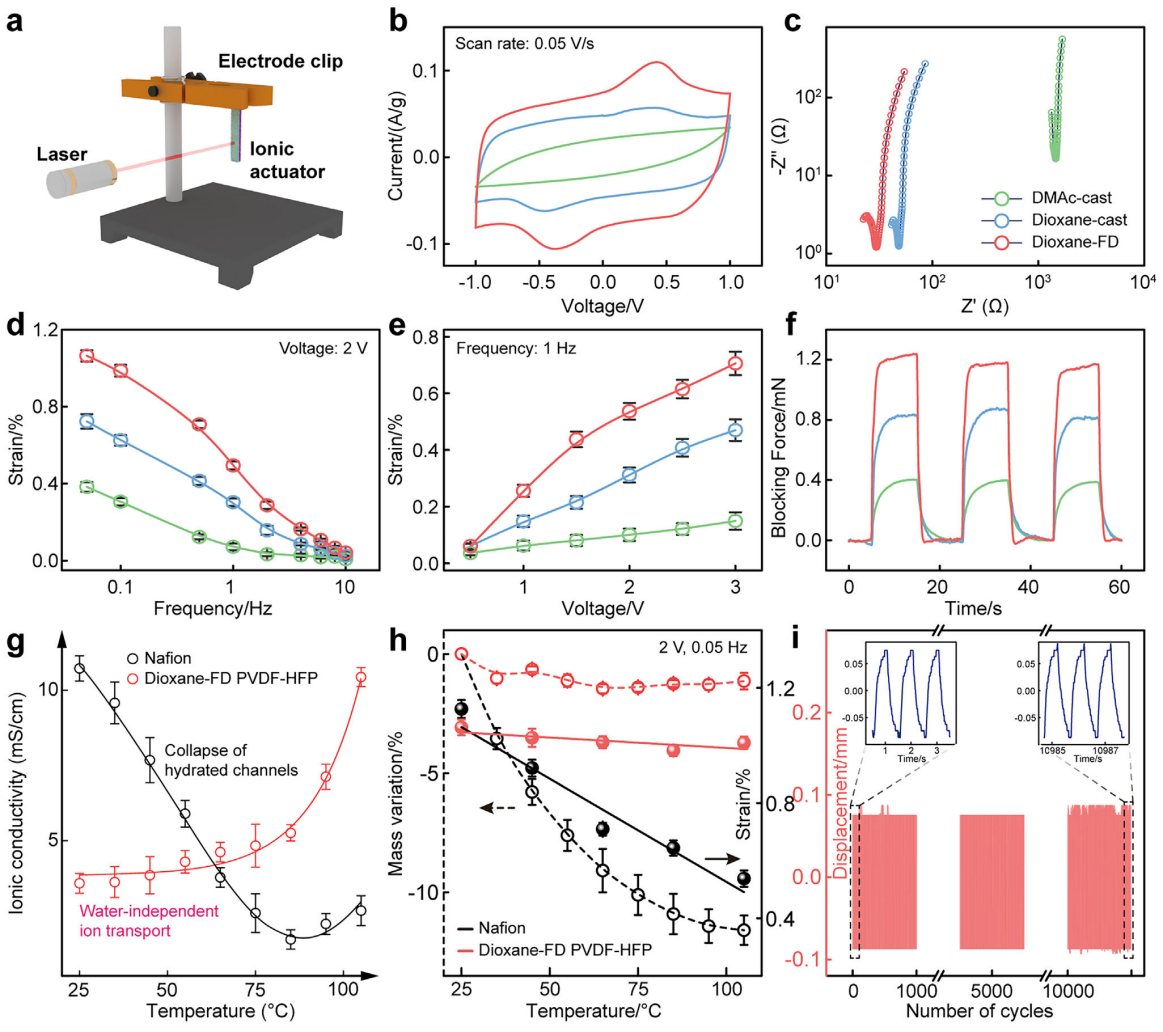
Comparative performance of PVDF‐HFP based ionic actuators. (a) Schematic of the experimental setup used for actuator displacement measurements. Systematic comparison of ionic actuators fabricated from PVDF‐HFP films prepared via DMAc‐cast, Dioxane‐cast, and Dioxane‐FD processes in terms of cyclic voltammetry curves (b), electrochemical impedance spectra (c), voltage and frequency response (d,e), and blocking force (f), reveals that the anisotropic PVDF‐HFP electrolyte exhibits superior electrochemical and actuation performance. (g,h) Thermal stability of Nafion and Dioxane‐FD PVDF‐HFP electrolytes. Water evaporation in Nafion at elevated temperatures reduces ionic conductivity and device performance, whereas the hydration‐independent, anisotropic freeze‐dried PVDF‐HFP maintains stable conductivity and actuation strain, demonstrating superior environmental robustness. i) Long‐term cycling performance of the actuator based on Dioxane‐FD PVDF‐HFP, showing stable displacement over more than 10 000 cycles (insets: enlarged views of selected cycles).

Beyond the actuation performance, the environmental stability of the device is further examined using a widely studied Nafion‐based ionic actuator as a benchmark. Before the temperature‐dependent measurements, the Nafion membrane was equilibrated with ambient air. Accordingly, its initial ionic conductivity of approximately 10 mS/cm corresponds to a partially hydrated state. Because ion transport in Nafion relies on dynamic hydrated channels, which collapse upon water evaporation at elevated temperatures, leading to a marked decline in ionic conductivity. Although Nafion‐based actuators exhibit slightly higher strain at room temperature, their performance deteriorates rapidly with increasing temperature. In contrast, Dioxane‐FD PVDF‐HFP with oriented channels maintains more stable ionic conductivity over a wide temperature range, and its intrinsically hydrophobic nature renders it insensitive to ambient temperature and humidity, thereby demonstrating superior environmental robustness (Figure 3 g,h). Further detailed comparisons are provided in Figures S13–S15. Furthermore, long‐term cycling tests show that the actuator based on the Dioxane‐FD PVDF‐HFP presents stable displacement over more than 10 000 cycles without noticeable degradation (Figure 3i), confirming its excellent durability.

### 2.4 Composition Optimization of Freeze‐Dried PVDF‐HFP Electrolytes

The freeze‐drying process is strongly affected by several parameters, including temperature, duration, concentration, and solvent content, all of which critically influence the morphology of the resulting electrolytes, thereby impacting the performance of ionic actuators. Given the high sensitivity of device performance to the concentration of ionic liquid (EMIM: BF_4_), this section systematically elucidates the effects of PVDF‐HFP and ionic liquid concentrations on film morphology. The influence of other parameters and the corresponding optimization analyses are provided in Figures S16–S20. Figure 4b summarizes the morphology evolution as a function of PVDF‐HFP and ionic liquid concentrations. Excessive ionic‐liquid loading compromised the continuity and structural integrity of the PVDF‐HFP framework, leading to the progressive collapse of the aligned porous channels and tearing of the freeze‐dried films. For instance, at a polymer load of 9 wt%, the resulting film exhibits no discernible porous structure when the ionic liquid ratio reaches 4.0, resembling a film obtained by casting. Here, the ionic liquid ratio refers to the mass ratio of the ionic liquid to the PVDF‐HFP polymer. Similarly, elevated PVDF‐HFP content reduces the solvent concentration, accelerating crystallization during freezing and resulting in fine, densely packed pores that inhibit the formation of continuous crystal channels and ultimately impede the establishment of oriented channels. Corresponding SEM analysis further reveals that at a polymer concentration of 15 wt% and an ionic liquid ratio of 1.5, the pore size is markedly reduced, accompanied by a clear decline in the anisotropic alignment of the porous structure. Our results suggest that an optimized electrolyte morphology, characterized by a well‐aligned porous network, could be achieved at a polymer concentration of 9 wt% and an ionic liquid ratio of 1.5.

**FIGURE 4:**
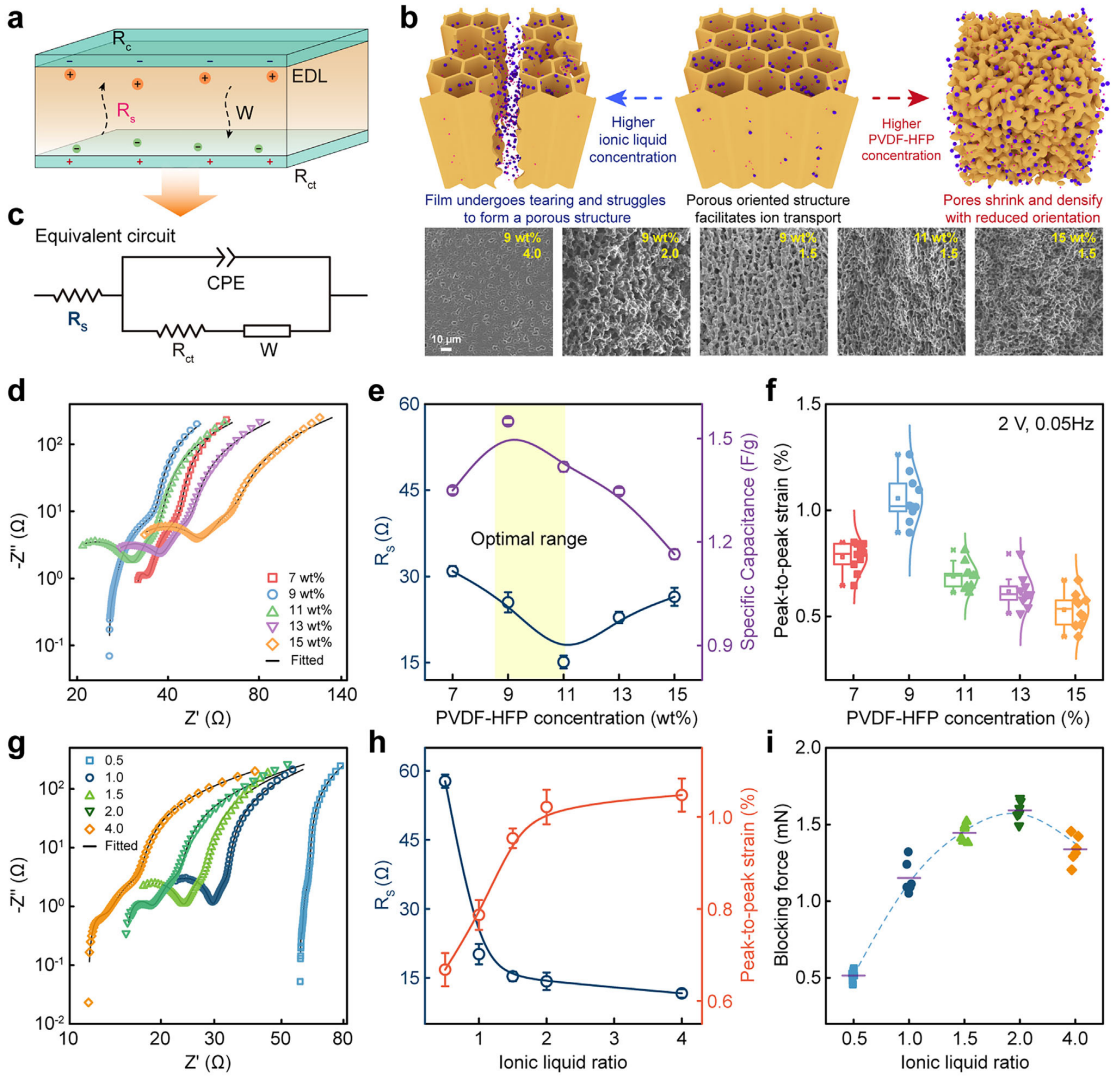
Electrolyte composition optimization and its impact on actuator performance. (a) Schematic illustration of the ionic actuator. (b) Structural evolution of freeze‐dried PVDF‐HFP based electrolytes with varying polymer and ionic liquid concentrations. Cross‐sectional SEM images indicate that excessive ionic liquid content compromises film integrity and causes tearing, while higher PVDF‐HFP concentrations markedly reduce pore size and disrupt channel alignment. An optimized composition (9 wt% PVDF‐HFP with an ionic liquid ratio of 1.5) yields a well‐defined, oriented porous structure. (c) Equivalent circuit of the ionic actuator. Electrochemical impedance spectrum (d), bulk ionic resistance (*R*s) and specific capacitance (e), and peak‐to‐peak strain (f) data for devices with varying PVDF‐HFP concentrations indicate that the 9 wt% formulation delivers optimal performance, consistent with the above structure analysis. With respect to different ionic liquid contents, electrochemical impedance spectra (g), bulk ionic resistance and strain (h), and blocking force (i) analysis show that increasing ionic liquid content enhances strain due to improved ion mobility, whereas the accompanying decrease in Young's modulus leads to a non‐monotonic trend in blocking force, which peaks at a ratio of 2.0.

Following the structural characterization, actuator performance is systematically evaluated to elucidate the impact of morphological changes on its functionality. An equivalent circuit model is proposed to further evaluate its electrochemical behavior (Figure 4c), where *R*_S_ is the bulk ionic resistance of the electrolyte, *R*_ct_ represents the electrode‐electrolyte interfacial resistance, and the constant phase element (CPE) accounts for the interfacial electric double‐layer capacitance (EDL). A Warburg element is further introduced to capture the low‐frequency ionic diffusion response. Figure 4d–f present the EIS, *R*_S_, specific capacitance, and strain output of actuators fabricated with PVDF‐HFP concentrations ranging from 7 to 15 wt%. Although the 9 wt% PVDF‐HFP film exhibits slightly higher *R*_S_ compared to the 11 wt% sample, it achieves the highest specific capacitance (∼1.55 F/g), likely due to more favorable ion‐transport channels formed by its oriented porous structure, as revealed by SEM analysis. Consequently, the actuator based on this composition exhibits the largest strain performance. Figure 4g–i illustrate the performance variations with different ionic liquid contents (ratios of 0.5, 1.0, 1.5, 2.0, and 4.0 relative to polymers). Both strain and blocking force increase markedly with rising ionic liquid content; however, beyond a ratio of 2.0, the strain enhancement becomes marginal, indicating ionic liquid saturation within the film. Meanwhile, the blocking force begins to decline, which may be attributed to a reduction in the film's Young's modulus caused by the excessive ionic liquid content. Overall, to achieve both high strain and sufficient force for practical applications, the optimal freeze‐drying formulation should contain ∼ 9 wt% PVDF‐HFP and an ionic liquid ratio between 1.5 and 2.0.

### 2.5 Versatile Applications of Ionic Actuators

To prove the practical applicability of the developed ionic actuator, its performance is examined in three representative scenarios: object grasping, steerable navigation, and integration into untethered soft robotics. Figure 5a compares the grasping capability of actuators fabricated with three different electrolytes. The actuator based on the DMAc‐cast PVDF‐HFP fails to grasp the object due to insufficient blocking force. Although the Nafion‐based actuator can initially lift the object, its performance deteriorates at elevated temperature (∼50°C), eventually leading to gripping failure. Conversely, the actuator employing the Dioxane‐FD PVDF‐HFP reliably grasps the objects while maintaining stable operation under the high‐temperature conditions, highlighting its thermal stability (Movie S2). Furthermore, its capability to manipulate small objects is demonstrated, enabling effective horizontal grasping and release, indicating the stiffness of the freeze‐dried electrolyte actuator, underscoring its potential for multidirectional operations in micromanufacturing and precision assembly (Figure S21). In addition, the fabricated ionic actuator is integrated onto the tip of a zebra guidewire to preliminarily explore its ability for steerable navigation. As shown in Figure 5b, the guidewire could traverse a set of rings under a low driving voltage of 2 V. Although the actuation is limited to the distal tip and yields a smaller bending angle (∼40°) than conventional magnetically actuated guidewires, it eliminates the need for bulky external magnetic systems, offering a distinct advantage for low‐power miniaturized operation (Movie S3).

**FIGURE 5:**
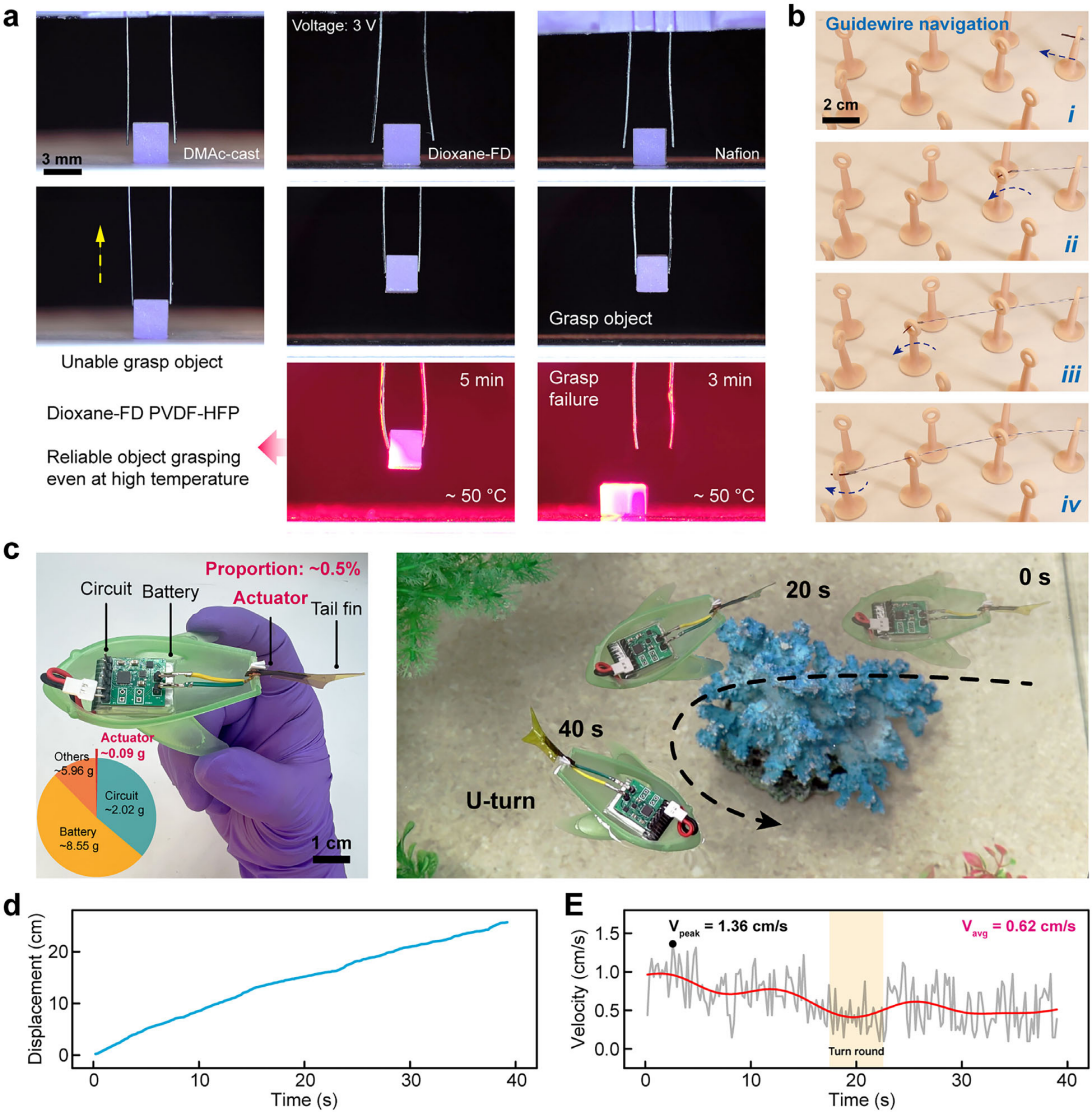
Versatile ionic actuator applications: from gripping to robotic propulsion. (a) Comparison of the grasping capability of ionic actuators based on DMAc‐cast PVDF‐HFP, Dioxane‐FD PVDF‐HFP, and Nafion electrolytes. The former fails to grasp the object (a cube of 3 mm and ∼ 5 mg) due to insufficient blocking force, and the Nafion actuator initially enables object grasping but loses performance at elevated temperatures of 50°C. By contrast, the Dioxane‐FD PVDF‐HFP actuator maintains stable grasping even under high temperatures, highlighting its superior environmental robustness. (b) Integration of an ionic actuator at the tip of a flexible zebra guidewire facilitates steerable navigation, showing promise for applications in minimally invasive interventions. (c) An untethered bio‐inspired robotic fish driven by a lightweight ionic actuator (∼0.5 % of total mass, 0.09 g). Displacement (d) and velocity (e) of robotic fish driven by a single ionic actuator, exhibiting efficient propulsion for soft aquatic robotics.

To further explore its applicability in bioinspired untethered robotics, the ionic actuator is integrated with a PCB control circuit and a battery module into a 3D‐printed soft robotic fish framework (Figure 5c). The actuator (1.8cm*0.5cm*0.2 mm) is attached to a tail fin cut from PET to generate sufficient propulsive force. The total mass of the battery (8.5 g), PCB board (2.0 g), and fish framework (6.0 g) is approximately 16.5 g. Notably, the ionic actuator itself weighs only 95 mg, accounting for merely ∼0.5% of the total system mass. The DC output from the battery is converted by the PCB board into a 3 V, 1 Hz AC signal, which drives the actuator to oscillate laterally, enabling forward motion and turning around (Figure S22 and Movie S4). Figure 5d,e present the displacement and velocity of the robotic fish. A maximum speed of 1.36 cm/s could be achieved, corresponding to ∼ 0.15 body length (BL) per second. The robotic fish prepared in this work is benchmarked against previously reported systems (Table S2). Although its speed is not the highest, its low contribution to the overall system mass represents a key advantage. It is capable of producing the necessary thrust for surface navigation of the aquatic robot, which confirms its strong suitability for use in lightweight and compact autonomous robotic platforms.

## 3 Conclusion

This study extends the ion transport framework in porous electrolytes by introducing an anisotropy factor, unifying the causal effects of random diffusion in disordered structures and directionally confined transport. The results show that oriented channels in anisotropic structures provide continuous, low‐resistance pathways, markedly enhancing ion transport along the channel direction. Electrolytes with oriented channels could be fabricated via directional freeze‐drying and exhibit pronounced directional dependence across multiple polymer systems, with substantially improved ion mobility along the aligned pathways compared to random structures. The resulting directional confinement enables simultaneous enhancement of strain and blocking force in ionic actuators, offering a route to mitigate the inherent trade‐off between these parameters. Moreover, unlike solvent‐dependent transport mechanisms, ion migration with channels is less sensitive to temperature variations, conferring excellent thermal stability and environmental robustness. Further tuning of polymer and ionic liquid concentrations enables optimization of electrolyte morphology, thereby maximizing device performance and supporting applications in robotic gripping and biomimetic systems.

## 4 Experimental Section

### 4.1 Materials

Poly (vinylidene fluoride‐co‐hexafluoropropylene) (PVDF‐HFP, average Mw: ∼455 000, Sigma–Aldrich) Poly (vinylidene fluoride) (PVDF, average Mw: ∼534 000, Sigma–Aldrich), Thermoplastic polyurethane (TPU, 75A), 1‐ethyl‐3‐methylimidazolium tetrafluoroborate (EMIM: BF_4_, 98%, Sigma–Aldrich), 1,4‐dioxane (>99.5%, Sigma–Aldrich), *N,N*‐dimethylacetamide (DMAc, anhydrous, >99.9%, Sigma–Aldrich), Poly(3,4‐ethylenedioxythiophene)‐poly(styrenesulfonate) (PEDOT: PSS, 1.3 wt % dispersion in H_2_O, conductive grade, Sigma–Aldrich), dimethylsulfoxide (DMSO, anhydrous, >99.9%, Sigma–Aldrich,) *t*‐Octylphenoxypolyethoxyethanol (Triton X‐100, Sigma–Aldrich), acetone (Tech grade, Aik Moh), and isopropyl alcohol (IPA, Tech grade, Aik Moh) are used as received without further purification. 20% Nafion dispersion solution and high‐purity single‐walled carbon nanotube (SWCNTs, XFS‐02, >90%, 5–30 µm in length, outside diameter in 1–2 nm, SSA >380 m^2^/g) are obtained from the Scientific Hub services, and Jiangsu XFNANO materials. Durapore PVDF membrane filters (0.22 µm pore size) are purchased from Merck Millipore, Sweden. Double‐deionized water was utilized throughout the study.

### 4.2 Electrolyte Preparation Procedure

As a representative example of oriented porous PVDF‐HFP films (9 wt% PVDF‐HFP, ionic liquid ratio 1.5), 1 g PVDF‐HFP is dissolved in 10 g 1,4‐dioxane under magnetic stirring in an oil bath at 70°C until a clear solution is obtained, followed by the addition of 1.5 g EMIM: BF_4_, which turned the solution milky white. A certain volume of the solution is then poured into a custom mold for directional freeze‐drying (featuring a copper base and PTFE walls), rapidly frozen in liquid nitrogen for ∼ 15 min, and immediately transferred to a freeze dryer (Labconco Corp., USA) for 12 h to obtain the anisotropic PVDF‐HFP electrolyte. Porous electrolytes based on TPU and PVDF are prepared following a similar protocol. An equal volume of commercial Nafion aqueous dispersion is loaded into the directional freeze‐drying mold, and the same procedure is repeated to acquire the freeze‐dried Nafion film. For casting TPU, PVDF, PVDF‐HFP, and Nafion films, the corresponding polymer solutions (dissolved in DMAc or 1,4‐dioxane) and commercial Nafion dispersion are poured into glass petri dishes and dried in an oven (90°C) until the solvent completely evaporates, forming uniform electrolyte films.

### 4.3 Electrode and Device Preparation Procedure

SWCNTs (10 mg) and Triton X‐100 (50 mg) are dispersed in 100 mL of deionized water via probe sonication in an ice bath. Subsequently, 5 wt% DMSO‐modified PEDOT: PSS solution is added and ultrasonicated to ensure homogeneity. The resulting dispersion is centrifuged at 4000 rpm for 30 min, and ∼ 80% of the supernatant is collected. Ionic liquid (10 mg) is added, followed by further sonication for uniform mixing. The mixture is vacuum filtered, washed sequentially with IPA/acetone (volume ratio 1: 1) and ample deionized water, then dried at 80°C for 4 h. The film is carefully peeled from the substrate and stacked with the electrolyte to form a sandwich structure. To ensure sufficient adhesion between the electrode and electrolyte, an appropriate amount of pristine electrolyte solution is applied between the layers. The assembly is clamped between glass plates using binder clips under moderate pressure for 2 h. After cooling to room temperature, the actuator is removed and cut into the desired shape for testing. Detailed methods are provided in the Supplementary Information.

### 4.4 Simulation Analysis of Ion Transport

Primary current distribution (cd) and transport of diluted species (tds) modules are employed to investigate ion mobility within the electrolyte. Three distinct structural models are constructed to represent PVDF‐HFP films using different solvents and processing methods: large and disordered pores; small and dense pores; and an oriented porous structure. Considering the substantial size disparities between EMIM^+^ and BF_4_^−^, the simulation focused primarily on the mobility of the cations. Assuming an initial cation accumulation near the electrolyte base, a ground potential was applied to the lower boundary and a negative voltage to the upper boundary to assess ion mobility behavior across distinct microstructures. In this work, three representative microstructural models were constructed: random structures containing large or small domains and an oriented porous structure. The anisotropy factor was defined as the ratio of the ion concentration changes measured on the top side of each model over the same time interval, as expressed by the following equation:

(1) $$\chi =\frac{\Delta c_{⊥}}{\Delta c_{∥}}$$

Building on this foundation, the solid mechanics module was integrated with a hygroscopic swelling interface to enable the multi‐physics coupling, allowing for the simulation of actuator deformation induced by ion mobility. A fixed boundary condition was applied to one end of the device to systematically evaluate its bending response under varying electrical stimuli and actuation durations (COMSOL Multiphysics simulation service provided by ZhaoRan Technology Co., Ltd.).

### 4.5 Characterization and Measurements

The porosity (*P*) of the PVDF‐HFP membranes is estimated using the apparent density method. The apparent density of the membrane is determined by measuring its mass, area, and thickness of the membrane and comparing it with the theoretical density of PVDF‐HFP to obtain the porosity:

(2) $$P=\left(1-\frac{\rho_{app}}{\rho_{th}}\right)\times 100\%$$

Mechanical properties and interfacial adhesion strength are evaluated via uniaxial tensile testing (Criterion Model 42, MTS), while surface and cross‐sectional morphologies were examined using field‐emission scanning electron microscopy (FESEM, JEOL 7600). X‐ray powder diffraction analysis is conducted by Bruker D8 Discover with nickel‐filtered Cu Kα radiation. The specific surface area is characterized by N_2_ adsorption–desorption isotherms (ASAP Tri‐star II 3020), and the orientation of different PVDF‐HFP films is analyzed by small‐angle x‐ray scattering (SAXS, Nanoinxider, Xenocs) with an exposure time of 20 min. For ionic conductivity measurements, the prepared membrane is sandwiched between two platinum electrodes and firmly secured using a clamp. Electrochemical impedance spectroscopy (EIS) was performed using an electrochemical workstation (PGSTAT302N), and the bulk resistance of the electrolyte was obtained by fitting the impedance spectra with an equivalent circuit model. The ionic conductivity was then calculated using the following equation:

(3) $$\sigma \left(S/cm\right)=\frac{d\left(cm\right)}{R\left(\Omega \right)\cdot A\left(cm^{2}\right)}$$

Here, *R*, *d*, and *A* represent the resistance, thickness and cross‐sectional area of the device, respectively. Ion mobility was evaluated from the transient current decay using the same setup. The membrane was first polarized at −1.5 V for a sufficient period to establish ion redistribution, followed immediately by the application of a +1.5 V bias. The characteristic migration time was defined as the time required for the transient current to decay by 90%, from which the apparent ion mobility was calculated. The specific formula is as follows:

(4) $$\mu \left(cm^{2}V^{-1}s^{-1}\right)\propto \frac{d^{2}\left(cm\right)}{V\left(V\right)\cdot t\left(s\right)}$$

Here, *V* refers to the applied voltage, *t* denotes the time of current decay, and *d* represents the thickness of the electrolyte. The specific capacitance is calculated using the following equation:

(5) $$C\left(F/g\right)=\frac{S}{2\cdot \nu \cdot \Delta V\cdot m}$$

Here, *S*, *v*, and Δ*V* are the area, scan rate, and potential window of the CV curve, while *m* represents the mass of the device.

The actuator (10 mm × 2 mm) is cut using a razor blade and securely clamped. A signal generator (JDS6600) applied the driving voltage, while a laser displacement sensor (Panasonic HG‐C1050) measured the displacement variation (*δ*) at a point approximately 3.5 mm (*L*) from the fixed end. A force gauge (FUTEK, USB220) was utilized to measure the blocking force, and the strain is then estimated using the following equation:

(6) $$\varepsilon \left(\%\right)=\frac{2d\delta }{L^{2}+\delta^{2}}$$

Here, *d* is the thickness of the actuator.

### 4.6 Variable‐Temperature Grasping Test

The ionic actuator is mounted onto a custom‐designed adapter prepared by 3D printing (Photo Mono 4, Anycubic), and subsequently integrated onto a robotic manipulator (Ufactory Lite 6). The actuator is electrically driven, while the manipulator provides controlled positioning and motion, enabling object grasping and transport. The target object is a cubic specimen with a side length of 3 mm and a mass of ∼5 mg. After stable grasping is achieved, localized heating is applied using an infrared irradiation source positioned adjacent to the gripping region to evaluate the thermal tolerance of the actuator.

### 4.7 Guidewire Navigation

The ionic actuator is integrated at the distal tip of a zebra guidewire, with copper wire connected to a power supply to enable electrically induced bending. During operation, the guidewire is advanced by external mechanical pushing from the proximal end, while the actuator provides directional control at the distal tip. By coordinating distal bending with forward motion, the guidewire is guided through confined pathways.

### 4.8 Fabrication of Untethered Robotic Fish

The framework of the robotic fish is fabricated with a 3D printer, and the printed circuit board is prepared by integrating a microcontroller (ATtiny2313A‐MUR, Microchip Technology), voltage regulator (TPS74529PQWDRVRQ1, Texas Instruments), H‐bridge motor (DRV8837DSGT, Texas Instruments), and capacitors (C0805C104K5RAC7800, KEMET). The tail fin is fabricated by cutting ∼ 100 µm‐thick PET film and is attached to the actuator using double‐sided adhesive tape. External programs could be uploaded to the microcontroller via the ISP interface on the Arduino board, enabling the conversion of the battery (3.7 V, 350 mA·h, YJ 702030) to a 3 V, 1 Hz AC signal for driving the locomotion of the robotic fish.

## Author Contributions

**Yufei Zhang**: conceptualization, investigation, methodology, writing − review and editing, writing − original draft, formal analysis, resources, and validation. **Qiuchun Lu**: methodology, software. **Adit Gupta**: methodology, visualization, software. **Sen Wang**: conceptualization, writing − review and editing. **Xueyang Wang**: methodology, conceptualization, writing − review and editing. **Jian Yu**: conceptualization, methodology. **Xuanyi Hu**: methodology, data curation. **Daniel Mandler**: conceptualization, funding acquisition, investigation, and writing − review and editing. **Pooi See Lee**: conceptualization, investigation, writing − review and editing, supervision, project administration, and funding acquisition.

## Conflicts of Interest

The authors declare no conflicts of interest.

## Supporting information




**Supporting File 1**: adma75384‐sup‐0001‐SuppMat.docx.

**Supporting File 2**: adma75384‐sup‐0002‐Movie1.mp4.

**Supporting File 3**: adma75384‐sup‐0003‐Movie2.mp4.

**Supporting File 4**: adma75384‐sup‐0004‐Movie3.mp4.

**Supporting File 5**: adma75384‐sup‐0005‐Movie4.mp4.

## Data Availability

The data that supports the findings of this study are available from the corresponding author upon reasonable request

## References

[1] R. H. Baughman , “Playing Nature's Game With Artificial Muscles,” Science 5718, no. 308 (2005): 63–65, 10.1126/science.1099010. 15802593

[2] C. Kaspar , B. J. Ravoo , W. G. van der Wiel , S. V. Wegner , and W. H. P. Pernice , “The Rise of Intelligent Matter,” Nature 7863, no. 594 (2021): 345–355, 10.1038/s41586-021-03453-y. 34135518

[3] W. Hu , G. Z. Lum , M. Mastrangeli , and M. Sitti , “Small‐Scale Soft‐Bodied Robot With Multimodal Locomotion,” Nature 7690, no. 554 (2018): 81–85, 10.1038/nature25443. 29364873

[4] D. Rus and M. T. Tolley , “Design, Fabrication and Control of Soft Robots,” Nature 7553, no. 521 (2015): 467–475, 10.1038/nature14543. 26017446

[5] L. Hines , K. Petersen , G. Z. Lum , and M. Sitti , “Soft Actuators for Small‐Scale Robotics,” Advanced Materials 13, no. 29 (2017): 1603483, 10.1002/adma.201603483. 28032926

[6] C. Christianson , N. N. Goldberg , D. D. Deheyn , S. Cai , and M. T. Tolley , “Translucent Soft Robots Driven by Frameless Fluid Electrode Dielectric Elastomer Actuators,” Science Robotics 17, no. 3 (2018): aat1893, 10.1126/scirobotics.aat1893. 33141742

[7] S. Wang , B. Huang , D. McCoul , M. Li , L. Mu , and J. Zhao , “A Soft Breaststroke‐Inspired Swimming Robot Actuated by Dielectric Elastomers,” Smart Materials and Structures 4, no. 28 (2019): 045006, 10.1088/1361-665X/ab0a7a.

[8] G. Li , X. Chen , F. Zhou , et al., “Self‐Powered Soft Robot in the Mariana Trench,” Nature 7848, no. 591 (2021): 66–71, 10.1038/s41586-020-03153-z. 33658693

[9] L. Shi , R. Yang , S. Lu , et al., “Dielectric Gels With Ultra‐High Dielectric Constant, Low Elastic Modulus, and Excellent Transparency,” NPG Asia Materials 8, no. 10 (2018): 821–826, 10.1038/s41427-018-0077-7.

[10] T. Li , G. Li , Y. Liang , et al., “Fast‐Moving Soft Electronic Fish,” Science Advances 4, no. 3 (2017): 1602045, 10.1126/sciadv.1602045. PMC5381956 28435879

[11] X. Gao , J. Yang , J. Wu , et al., “Piezoelectric Actuators and Motors: Materials, Designs, and Applications,” Advanced Materials Technologies 1, no. 5 (2020): 1900716, 10.1002/admt.201900716.

[12] Y. Wang , J. Deng , S. Ma , W. Chen , S. Zhang , and Y. Liu , “Tunable Imaging Distance Focusing Module Directly Driven by a Thin‐Plate Piezoelectric Actuator,” Advanced Functional Materials 8, no. 35 (2025): 2415918, 10.1002/adfm.202415918.

[13] X. Tian , Y. Guo , J. Zhang , O. M. Ivasishin , J. Jia , and J. Yan , “Fiber Actuators Based on Reversible Thermal Responsive Liquid Crystal Elastomer,” Small 24, no. 20 (2024): 2306952, 10.1002/smll.202306952. 38175860

[14] H. Ryu , J. W. Song , H. Luan , et al., “Materials and Device Designs for Wireless Monitoring of Temperature and Thermal Transport Properties of Wound Beds during Healing,” Advanced Healthcare Materials 5, no. 13 (2024): 2302797, 10.1002/adhm.202302797. PMC11468708 37983897

[15] S. Wu , T. Zhao , Y. Zhu , and G. H. Paulino , “Modular Multi‐Degree‐of‐Freedom Soft Origami Robots With Reprogrammable Electrothermal Actuation,” Proceedings of the National Academy of Sciences 20, no. 121 (2024): 2322625121, 10.1073/pnas.2322625121. PMC11098090 38709915

[16] M. D. Bartlett , N. Kazem , M. J. Powell‐Palm , et al., “High Thermal Conductivity in Soft Elastomers With Elongated Liquid Metal Inclusions,” Proceedings of the National Academy of Sciences 9, no. 114 (2017): 2143–2148, 10.1073/pnas.1616377114. PMC5338550 28193902

[17] Y. Ohm , C. Pan , M. J. Ford , X. Huang , J. Liao , and C. Majidi , “An Electrically Conductive Silver–Polyacrylamide–Alginate Hydrogel Composite for Soft Electronics,” Nature Electronics 3, no. 4 (2021): 185–192, 10.1038/s41928-021-00545-5.

[18] G. Gu , M. Schmid , P.‐W. Chiu , et al., “V_2_O_5_ Nanofibre Sheet Actuators,” Nature Materials 5, no. 2 (2003): 316–319, 10.1038/nmat880. 12704380

[19] R. H. Baughman , C. Cui , A. A. Zakhidov , et al., “Carbon Nanotube Actuators,” Science 5418, no. 284 (1999): 1340–1344. 10.1126/science.284.5418.1340 10334985

[20] V. H. Ebron , Z. Yang , D. J. Seyer , et al., “Fuel‐Powered Artificial Muscles,” Science 5767, no. 311 (2006): 1580–1583, 10.1126/science.1120182. 16543453

[21] S. Iamsaard , S. J. Aßhoff , B. Matt , et al., “Conversion of Light into Macroscopic Helical Motion,” Nature Chemistry 3, no. 6 (2014): 229–235, 10.1038/nchem.1859. 24557138

[22] Z. Pei , Y. Yang , Q. Chen , E. M. Terentjev , Y. Wei , and Y. Ji , “Mouldable Liquid–Crystalline Elastomer Actuators With Exchangeable Covalent Bonds,” Nature Materials 1, no. 13 (2014): 36–41, 10.1038/nmat3812. 24292422

[23] C. S. Haines , M. D. Lima , N. Li , et al., “Artificial Muscles From Fishing Line and Sewing Thread,” Science 6173, no. 343 (2014): 868–872, 10.1126/science.1246906. 24558156

[24] Q. Shen , T. Wang , J. Liang , and L. Wen , “Hydrodynamic Performance of a Biomimetic Robotic Swimmer Actuated by Ionic Polymer–metal Composite,” Smart Materials and Structures 7, no. 22 (2013): 075035, 10.1088/0964-1726/22/7/075035.

[25] Z. Chen , T. I. Um , and H. Bart‐Smith , “Bio‐Inspired Robotic Manta Ray Powered by Ionic Polymer–Metal Composite Artificial Muscles,” International Journal of Smart and Nano Materials 4, no. 3 (2012): 296–308, 10.1080/19475411.2012.686458.

[26] Z. Chen , S. Shatara , and X. Tan , “Modeling of Biomimetic Robotic Fish Propelled by an Ionic Polymer–Metal Composite Caudal Fin,” IEEE/ASME Transactions on Mechatronics 3, no. 15 (2010): 448–459, 10.1109/TMECH.2009.2027812.

[27] Y. Zhang , A. Gupta , Q. Lu , et al., “Optimization of Core–Shell Ternary Electrodes for High‐Performance Ionic Actuator in Soft Gripper,” Advanced Materials 26, no. 37 (2025): 2503297, 10.1002/adma.202503297. PMC12232216 40331483

[28] V. H. Nguyen , J. Kim , R. Tabassian , et al., “Electroactive Artificial Muscles Based on Functionally Antagonistic Core–Shell Polymer Electrolyte Derived from PS‐ b ‐PSS Block Copolymer,” Advanced Science 5, no. 6 (2019): 1801196, 10.1002/advs.201801196. PMC6402454 30886790

[29] G. Wu , Y. Hu , Y. Liu , et al., “Graphitic Carbon Nitride Nanosheet Electrode‐Based High‐Performance Ionic Actuator,” Nature Communications 1, no. 6 (2015): 7258, 10.1038/ncomms8258. PMC4458862 26028354

[30] Z. Zhu , H. Chen , L. Chang , and B. Li , “Dynamic Model of Ion and Water Transport in Ionic Polymer–Metal Composites,” AIP Advances 4, no. 1 (2011): 040702, 10.1063/1.3668286.

[31] E. Shoji and D. Hirayama , “Effects of Humidity on the Performance of Ionic Polymer−Metal Composite Actuators: Experimental Study of the Back‐Relaxation of Actuators,” Journal of Physical Chemistry B 41, no. 111 (2007): 11915–11920, 10.1021/jp074611q. 17887668

[32] Z. Zhu , L. Chang , K. Asaka , et al., “Comparative Experimental Investigation on the Actuation Mechanisms of Ionic Polymer–Metal Composites With Different Backbones and Water Contents,” Journal of Applied Physics 12, no. 115 (2014): 124903, 10.1063/1.4869537.

[33] M. Shahinpoor and K. J. Kim , “Mass Transfer Induced Hydraulic Actuation in Ionic Polymer–Metal Composites,” Journal of Intelligent Material Systems and Structures 6, no. 13 (2002): 369–376, 10.1177/104538902761696715.

[34] J. Ko , D. Kim , Y. Song , et al., “Electroosmosis‐Driven Hydrogel Actuators Using Hydrophobic/Hydrophilic Layer‐By‐Layer Assembly‐Induced Crack Electrodes,” ACS Nano 9, no. 14 (2020): 11906–11918, 10.1021/acsnano.0c04899. 32885947

[35] J. Ko , C. Kim , D. Kim , et al., “High‐Performance Electrified Hydrogel Actuators Based on Wrinkled Nanomembrane Electrodes for Untethered Insect‐Scale Soft Aquabots,” Science Robotics 71, no. 7 (2022): abo6463, 10.1126/scirobotics.abo6463. 36288271

[36] J. Foroughi , G. M. Spinks , and G. G. Wallace , “A Reactive Wet Spinning Approach to Polypyrrole Fibres,” Journal of Materials Chemistry 17, no. 21 (2011): 6421–6426, 10.1039/c0jm04406g.

[37] H. Choi , Y. Kim , S. Kim , et al., “Ions‐Silica Percolated Ionic Dielectric Elastomer Actuator for Soft Robots,” Advanced Science 32, no. 10 (2023): 2303838, 10.1002/advs.202303838. PMC10646257 37792271

[38] S. Cao , J. Aimi , and M. Yoshio , “Electroactive Soft Actuators Based on Columnar Ionic Liquid Crystal/Polymer Composite Membrane Electrolytes Forming 3D Continuous Ionic Channels,” ACS Applied Materials & Interfaces 38, no. 14 (2022): 43701–43710, 10.1021/acsami.2c11029. 36044399

[39] S. Y. Kim , Y. Kim , C. Cho , et al., “Deformable Ionic Polymer Artificial Mechanotransducer With an Interpenetrating Nanofibrillar Network,” ACS Applied Materials & Interfaces 32, no. 11 (2019): 29350–29359, 10.1021/acsami.9b10499. 31313573

[40] F. Hu , Y. Xue , J. Xu , and B. Lu , “PEDOT‐Based Conducting Polymer Actuators,” Frontiers in Robotics and AI 6 (2019): 114, 10.3389/frobt.2019.00114. PMC7805747 33501129

[41] Y. Li , W. Liu , X. Gao , et al., “Carbon Nanomaterials‐PEDOT: PSS Based Electrochemical Ionic Soft Actuators: Recent Development in Design and Applications,” Sensors and Actuators A: Physical 354 (2023): 114277, 10.1016/j.sna.2023.114277.

[42] M. Garai , M. Mahato , S. Nam , et al., “Metal Organic Framework‐MXene Nanoarchitecture for Fast Responsive and Ultra‐Stable Electro‐Ionic Artificial Muscles,” Advanced Functional Materials 10, no. 33 (2023): 2212252, 10.1002/adfm.202212252.

[43] G. Wu , X. Wu , Y. Xu , et al., “High‐Performance Hierarchical Black‐Phosphorous‐Based Soft Electrochemical Actuators in Bioinspired Applications,” Advanced Materials 25, no. 31 (2019): 1806492, 10.1002/adma.201806492. 31012167

[44] D. Guo , Y. Han , J. Huang , et al., “Hydrophilic Poly(vinylidene Fluoride) Film With Enhanced Inner Channels for Both Water‐ and Ionic Liquid‐Driven Ion‐Exchange Polymer Metal Composite Actuators,” ACS Applied Materials & Interfaces 2, no. 11 (2019): 2386–2397, 10.1021/acsami.8b18098. 30604952

[45] M. D. Bennett and D. J. Leo , “Ionic Liquids as Stable Solvents for Ionic Polymer Transducers,” Sensors and Actuators A: Physical 1, no. 115 (2004): 79–90, 10.1016/j.sna.2004.03.043.

[46] M. Armand , F. Endres , D. R. MacFarlane , H. Ohno , and B. Scrosati , “Ionic‐liquid Materials for the Electrochemical Challenges of the Future,” Nature Materials 8, no. 8 (2009): 621–629, 10.1038/nmat2448. 19629083

[47] I. Takeuchi , K. Asaka , K. Kiyohara , et al., “Electromechanical Behavior of Fully Plastic Actuators Based on Bucky Gel Containing Various Internal Ionic Liquids,” Electrochimica Acta 6, no. 54 (2009): 1762–1768, 10.1016/j.electacta.2008.10.007.

[48] A. Kusoglu and A. Z. Weber , “New Insights Into Perfluorinated Sulfonic‐Acid Ionomers,” Chemical Reviews 3, no. 117 (2017): 987–1104, 10.1021/acs.chemrev.6b00159. 28112903

[49] K. A. Mauritz and R. B. Moore , “State of Understanding of Nafion,” Chemical Reviews 10, no. 104 (2004): 4535–4586, 10.1021/cr0207123. 15669162

[50] J. Ru , Y. Wang , L. Chang , H. Chen , and D. Li , “Preparation and Characterization of Water‐Soluble Carbon Nanotube Reinforced Nafion Membranes and So‐Based Ionic Polymer Metal Composite Actuators,” Smart Materials and Structures 9, no. 25 (2016): 095006, 10.1088/0964-1726/25/9/095006.

[51] V. H. Nguyen , S. Oh , M. Mahato , et al., “Functionally Antagonistic Polyelectrolyte for Electro‐Ionic Soft Actuator,” Nature Communications 1, no. 15 (2024): 435, 10.1038/s41467-024-44719-z. PMC10781978 38200009

[52] J.‐H. Jung , J.‐H. Jeon , V. Sridhar , and I.‐K. Oh , “Electro‐Active Graphene–Nafion Actuators,” Carbon 4, no. 49 (2011): 1279–1289, 10.1016/j.carbon.2010.11.047.

[53] Y. Lian , Y. Liu , T. Jiang , J. Shu , H. Lian , and M. Cao , “Enhanced Electromechanical Performance of Graphite Oxide–Nafion Nanocomposite Actuator,” Journal of Physical Chemistry C 21, no. 114 (2010): 9659–9663, 10.1021/jp101337h.

[54] R. Liu , Z. Li , H. Zhao , et al., “Dual‐Stimuli‐Responsive Covalent Organic Framework‐Liquid Crystal Polymer Smart Actuator,” Advanced Functional Materials 15, no. 35 (2025): 2418627, 10.1002/adfm.202418627.

[55] G. Chen , L. Chen , N. Li , et al., “Salt‐Assisted Fabrication of a Water‐Based Covalent Organic Framework Ink and Its Hybrid Films for Photothermal Actuators,” ACS Applied Materials & Interfaces 30, no. 15 (2023): 36804–36810, 10.1021/acsami.3c06435. 37478481

[56] A. Maziz , C. Plesse , C. Soyer , et al., “Demonstrating kHz Frequency Actuation for Conducting Polymer Microactuators,” Advanced Functional Materials 30, no. 24 (2014): 4851–4859, 10.1002/adfm.201400373.

[57] F. Vidal , C. Plesse , P.‐H. Aubert , et al., “Poly(3,4‐ethylenedioxythiophene)‐Containing Semi‐Interpenetrating Polymer Networks: A Versatile Concept for the Design of Optical or Mechanical Electroactive Devices,” Polymer International 3, no. 59 (2010): 313–320, 10.1002/pi.2772.

[58] N. Festin , A. Maziz , C. Plesse , D. Teyssié , C. Chevrot , and F. Vidal , “Robust Solid Polymer Electrolyte for Conducting IPN Actuators,” Smart Materials and Structures 10, no. 22 (2013): 104005, 10.1088/0964-1726/22/10/104005.

[59] F. Yu , J.‐H. Ciou , S. Chen , et al., “Ionic Covalent Organic Framework Based Electrolyte for Fast‐Response Ultra‐Low Voltage Electrochemical Actuators,” Nature Communications 1, no. 13 (2022): 390, 10.1038/s41467-022-28023-2. PMC8770580 35046389

[60] J. Fuller , A. C. Breda , and R. T. Carlin , “Ionic Liquid–polymer Gel Electrolytes From Hydrophilic and Hydrophobic Ionic Liquids,” Journal of Electroanalytical Chemistry 1, no. 459 (1998): 29–34, 10.1016/S0022-0728(98)00285-X.

[61] T. Fukushima , K. Asaka , A. Kosaka , and T. Aida , “Fully Plastic Actuator through Layer‐by‐Layer Casting With Ionic‐Liquid‐Based Bucky Gel,” Angewandte Chemie International Edition 16, no. 44 (2005): 2410–2413, 10.1002/anie.200462318. 15761901

[62] K. Aoki , A. Sugawara‐Narutaki , Y. Doi , and R. Takahashi , “Structure and Rheology of Poly(vinylidene difluoride‐co‐hexafluoropropylene) in an Ionic Liquid: The Solvent Behaves as a Weak Cross‐Linker through Ion–Dipole Interaction,” Macromolecules 13, no. 55 (2022): 5591–5600, 10.1021/acs.macromol.2c00485.

[63] U. Raza , S. Oh , R. Tabassian , M. Mahato , V. H. Nguyen , and I.‐K. Oh , “Micro‐Structured Porous Electrolytes for Highly Responsive Ionic Soft Actuators,” Sensors and Actuators B: Chemical 352 (2022): 131006, 10.1016/j.snb.2021.131006.

[64] W. Shasha , M. Penghui , L. Yang , et al., “Preparation of Polyvinylidene Fluoride–Hexafluoropropylene Electrolyte Membrane by Electrospinning Method for Electrochemical Actuators,” Acta Chimica Sinica 6, no. 82 (2024): 570–576.

[65] L. Wang , Y. Xiao , S. Guan , et al., “From Membranes to Electrodes: Aligned Membrane Electrode Assemblies for Ion‐Exchange Membrane Fuel Cells and Water Electrolyzers,” Electrochemical Energy Reviews 1, no. 9 (2026): 4, 10.1007/s41918-025-00269-6.

[66] Y. Ren , L. He , and H. Xia , “Water Insoluble Polymeric Materials With Well‐Oriented Structures Prepared by Ice‐Templating: A Mini‐Review,” Journal of Polymer Science 14, no. 62 (2024): 3156–3170, 10.1002/pol.20230500.

[67] B. Tjaden , S. J. Cooper , D. J. L. Brett , D. Kramer , and P. R. Shearing , “On the Origin and Application of the Bruggeman Correlation for Analysing Transport Phenomena in Electrochemical Systems,” Current Opinion in Chemical Engineering 12 (2016): 44–51, 10.1016/j.coche.2016.02.006.

[68] M. Alipour , Y. Li , H. Liu , and A. A. Pahlavan , “Diffusiophoretic Transport of Colloids in Porous media,” Science Advances 7, no. 12 (2026): ady9874, 10.1126/sciadv.ady9874. PMC12893296 41671380

