# Supplementary material for: Directional Confinement Governed Ion Transport for Efficient Actuation
Source: Adv Mater. 2026 Oct 15;38(63):e75384. doi: 10.1002/adma.75384 (PMC13755671; doi:10.1002/adma.75384)
Supplement: Supplementary file 1 [file ADMA-38-e75384-s004.docx]

Copyright WILEY-VCH Verlag GmbH & Co. KGaA, 69469 Weinheim, Germany, 2016.

Supporting Information

**Directional confinement governed ion transport for efficient actuation**

*Yufei Zhang, Qiuchun Lu, Adit Gupta, Sen Wang, Xueyang Wang, Jian Yu, Xuanyi Hu, Daniel Mandler, Pooi See Lee*

**Contents**

**A:** **Analysis of ion transport in porous electrolyte**

**B:** **Simulation of ion mobility and device bending**

**C:** **Comparison of PVDF-HFP electrolytes with different morphologies**

**D:** **Actuator fabrication and performance comparison with Nafion-based devices**

**E:** **Performance optimization of freeze-dried films**

**F:** **Multifunctional applications of the actuator**

**A: Analysis of ion transport in porous electrolyte**

The Nernst-Planck (NP) equation provides the fundamental framework for describing ion transport in electrochemical systems, and its complete form is given as follows.

 (1-1)

Where $d_{I}$ is the self-diffusion coefficient of the counter ion, $M_{I}$ is the molar weight of component *I*, and $\overline{\nu}$ denotes the convection velocity. Since the diffusion flux induced by the pressure gradient is typically negligible, the above equation could be further simplified as follows.

 (1-2)

The above equation could be further expanded into the following form^[1,2]^.

$J_{I}=-D_{I}\nabla c_{I}+\frac{F}{RT}z_{I}c_{I}\mu_{I}E$ (1-3)

Where $\mu_{I}$ is the ion mobility, and $E$ is the electric field. The relationship between ion mobility and diffusion coefficient is governed by the Nernst-Einstein relation:

$\mu_{I}=\frac{Z_{I}F}{RT}D_{I}$ (1-4)

For homogeneous media, the characteristic timescale of diffusion over a transport distance *L* is given by:

$t\sim\frac{L^{2}}{D_{I}}$ (1-5)

It is evident that as the transport distance increases, the efficiency of diffusion-driven transport decreases significantly. For a porous electrolyte, ion transport is additionally influenced by microstructure. In classical porous-media theory, the porosity ($\varepsilon$) determines the fraction of accessible volume for ion mobility, while the tortuosity ($\tau$) quantifies the increased path length due to the porous structure. The effective ion mobility could be modified as follows^[3]^:

$\mu_{eff}\propto\frac{D_{eff}}{k_{B}T}\propto\frac{\varepsilon}{\tau}$ (1-6)

This treatment is consistent with Bruggeman-type effective medium models; however, such relations are only valid for random and isotropic porous structures and do not account for directional-dependent arising from anisotropic geometries. To elucidate the role of tortuosity in diffusion-dominated transport, the effective transport path can be expressed as:

$L_{eff}=\tau L$ (1-7)

Substituting this path elongation into the diffusion timescale gives:

$t\sim\frac{L_{eff}^{2}}{D_{I}}=\frac{(\tau L)^{2}}{D_{I}}=\tau^{2}\frac{L^{2}}{D_{I}}$ (1-8)

This result suggests that, under a random diffusion approximation, tortuosity imposes a quadratic penalty on the transport timescale. Correspondingly, the effective mobility follows the scaling:

$\mu_{eff}\propto\frac{\varepsilon}{\tau^{2}}$ (1-9)

It should be noted that the $\tau^{2}$ dependence is not a universal relation, but rather a manifestation of a diffusion-limited scaling effect, where ion transport can be approximated as an elongated random walk. However, this tortuosity-based description is highly model-sensitive and becomes less reliable in complex or anisotropic structures. In this work, we focus on elucidating the directional disparity of ion transport within ordered anisotropic channels. Accordingly, an anisotropy factor, $\chi$, is introduced to quantify the preferential enhancement of ion transport along the aligned channel direction. In the simulations, $\chi$ is approximately based on the directional differences in concentration evolution.

$\chi=\frac{\Delta c_{\perp}}{\Delta c_{\parallel}}$ (1-10)

where $\Delta c_{\perp}$ and $\Delta c_{\parallel}$ denote the concentration variation measured along the through-plane and in-plane direction, respectively. A larger $\chi$ indicated stronger structural anisotropy and more pronounced directional transport. With the emergence of directional confinement, ion migration depends not only on porosity and tortuosity but also on preferential low-resistance pathways. In this intermediate regime, the effective mobility can be expressed as:

$\mu_{eff}\propto\frac{\varepsilon}{\tau}\cdot\chi$ (1-11)

When the oriented channels become continuous and dominant, the transport process transitions from highly tortuous stochastic motion to pathway-governed directional migration. In this regime, the influence of porosity diminishes, and the effective mobility along the aligned pathways is primarily dictated by their tortuosity.

$\mu_{eff}\propto\frac{\chi}{\tau}$ (1-12)

Overall, this framework extends the conventional porosity-tortuosity description by explicitly incorporating anisotropy as an additional governing parameter, thereby providing a mechanistic basis for understanding and designing efficient ion transport in channel-engineered porous electrolytes.

**B: Simulation of ion mobility and device bending**

**Figure S1. Simulated ion transport in the through-plane direction of different structures, showing that the directionally confined channels exhibit the fastest ion transport.**

**Figure S2. In-plane ion transport across different electrolyte structures, showing minimal variation due to the predominantly random and disordered morphology, while structures with large particles and high tortuosity exhibit reduced transport rates.**

**Figure S3. Simulated ion migration in various electrolytes and evaluated its bending performance (a). Bending behavior as a function of voltage (b) and time (c) is presented.**

In the simulation of actuator bending performance, ions are assumed to be initially uniformly distributed within the electrolyte, and the applied voltage induces concentration gradients that drive bending deformation. Results demonstrate that an oriented porous structure enhances ion mobility, leading to increased deformation. Figure S3b and S3c illustrate performance variations with voltage polarity and duration: voltage reversal induces ion redistribution and bending direction change, while extended voltage application intensifies concentration gradients, resulting in greater deformation.

**C: Comparison of PVDF-HFP electrolytes with different morphologies**

**Figure S4. Schematic of the preparation procedure of the Dioxane-FD PVDF-HFP films.**

Initially, PVDF-HFP is dissolved in 1,4-dioxane to form a clear, transparent solution. Upon the addition of the ionic liquid, the solution turns milky white. This mixture is then poured into a directional freeze-drying mold (featuring a copper base and PTFE walls) and immersed in liquid nitrogen, where the solvent crystallizes along the vertical direction. Finally, the frozen sample is subjected to freeze-drying for approximately 12 h.

**Figure S5. Characterization of three PVDF-HFP samples with distinct morphologies.** a) Cross-sectional SEM images of PVDF-HFP films prepared via DMAc-cast, Dioxane-cast, and Dioxane-FD. EDS mapping (b) and pore size distribution (c) of Dioxane-cast PVDF-HFP film. d-e) XRD patterns and SAXS curves of three films.

Figure S5a shows cross-sectional SEM images of PVDF-HFP films prepared via DMAc-cast, Dioxane-cast, and Dioxane-FD. The Dioxane-cast film shows improved density and uniformity relative to DMAc, whereas the Dioxane-FD film exhibits increased thickness and an oriented porous structure. EDS mapping confirms the homogeneous distribution of boron (B), indicating uniform dispersion of the ionic liquid (EMIM: BF_4_) in the electrolyte. (Figure S5b). BET analysis of Dioxane-FD PVDF-HFP reveals a porous network with pore sizes centered around 20 nm (Figure S5c). XRD and SAXS results (Figure S5d-e) further verify that the crystalline structure of PVDF-HFP is unaffected by processing conditions.

**Figure S6. Thermogravimetric analysis (TGA) (a), differential scanning calorimetry (DSC) (b), and dynamic mechanical analysis (DMA) of three PVDF-HFP films.**

The TGA results show that all three samples retain more than ~95% of their initial mass below 250 °C and exhibit similar multistep thermal decomposition behavior over approximately 270-510 °C. These results indicate that the different membrane fabrication methods have a limited influence on the overall thermal decomposition behavior of the electrolytes and that all three electrolytes exhibit good thermal stability within the intended operating temperature range of the devices. In contrast, the three samples exhibit distinctly different crystallization behavior. The DMAc-cast membrane shows a broad crystallization peak at approximately 70-75 °C, indicating that crystallization requires greater supercooling and occurs over a broader temperature range. Dioxane-FD and Dioxane-cast membranes exhibit sharper crystallization peaks at approximately 90 and 100 °C, respectively, indicating more concentrated crystallization at lower degrees of supercooling. DMA measurements further reveal the influence of different microstructures on the thermomechanical properties of the electrolytes. The storage moduli of DMAc-cast, Dioxane-cast, and Dioxane-FD decrease from approximately 8.2, 16, and 10.5 MPa at 25 °C to 2.0, 4.5, and 2.6 MPa at 100 °C, respectively. Although the introduction of porosity reduces the stiffness of the Dioxane-FD membrane relative to the Dioxane-cast membrane, its storage modulus remains higher than that of the DMAc-cast membrane with large domains. This indicates that the oriented porous structure formed by directional freeze-drying maintains sufficient thermomechanical integrity while providing continuous through-thickness ion transport pathways, thereby achieving a favorable balance between ion transport efficiency and structural stability.

**Figure S7.** **FTIR (a) and mechanical property analysis (b) of three PVDF-HFP films.**

In PVDF-HFP/ionic liquid composite films, potential interactions between the cations (EMIM^+^) of the ionic liquid and the polymer backbone may alter the polymer’s conformational structure. To investigate these interactions, FTIR spectra (500~3500 cm^-1^) are collected for films prepared with varying solvents and ionic liquid concentrations (Figure S7a). The numerical labels 0.5, 1.0, 1.5, 2.0, and 4.0 represent the mass ratio of EMIM: BF_4_ to PVDF-HFP; for example, 2.0 denotes 2.0 g of ionic liquid per gram of PVDF-HFP. The spectra could be divided into two regions: Region II features peaks attributed to the ethyl chains and imidazolium ring C-H stretching of the ionic liquid, along with polymer backbone vibrations; Region I corresponds to crystalline and amorphous phases of PVDF-HFP. As the ionic liquid content increases, characteristic peaks shift toward lower wavenumbers, suggesting an increased amorphous fraction due to polymer-ionic liquid interactions. A comparable shift is observed among the three different films at identical ionic liquid concentrations, with the Dioxane-FD film exhibiting the largest shift, indicating a higher ionic liquid content facilitated by its oriented porous structure. As shown in Figure S7b, increasing ionic liquid content reduces mechanical strength, with the dense Dioxane-cast film showing the highest robustness and the Dioxane-FD film the lowest Young’s modulus.

**Figure S8. Photographs (a), EIS data (b), thickness (c), and ionic conductivity (d) of three PVDF-HFP films at different ionic liquid concentrations.**

The PVDF-HFP films prepared via DMAc-cast, Dioxane-cast, and Dioxane-FD with different ionic liquid concentrations are shown in Figure S8a. The films fabricated via casting processes exhibit high optical transparency, whereas the Dioxane-FD films appear white. For all three sample types, increasing the ionic liquid content leads to a decline in film-forming quality, with evident fragmentation observed when the ionic liquid ratio reaches 4.0. Figures S8b-c present the impedance and thickness variation of films, respectively. DMAc-cast films show greater thickness than Dioxane-cast and Dioxane-FD films. This is likely attributed to the slow evaporation rate of DMAc, which can promote phase separation during the drying process, leading to less uniform and thicker films. In contrast, the low boiling point of 1,4-dioxane allows for rapid evaporation, reducing polymer aggregation and promoting uniform film formation, thus yielding thinner films. The impedance decreases significantly with increasing ionic liquid content in all samples. By integrating thickness and impedance data, the ionic conductivity of each film was calculated (Figure S8d). The Dioxane-FD films exhibited superior ionic conductivity relative to the other two, with values reaching approximately 10 mS/cm, owing to their well-aligned porous architecture that facilitates efficient ion transport.

**Figure S9. Analysis of ion mobility in the electrolytes.** In freeze-drying films, the through-plane mobility exceeds the in-plane value by more than a factor of two (a-b), whereas casting films exhibit nearly identical mobilities in both directions (c-d), demonstrating that directional confinement markedly enhances ion transport along the desired pathways.

**Figure S10. Universality of directional confined ion transport.** Freeze-dried TPU (a) and PVDF (b) films with oriented porous structure exhibit significantly enhanced ionic conductivity and mobility compared to their casting counterparts.

**D: Actuator fabrication and performance comparison with Nafion-based devices**

**Figure S11. Preparation and characterization of the electrode.** a) Schematic illustration of a ternary electrode using vacuum filtration. Optical photograph (b) and SEM image (c) of the ternary electrode, with the inset cross-sectional view revealing the layered fibrous structure. XRD pattern (d), mechanical properties (e), CV curve (f), and impedance data (g) indicate that the ternary electrode composed of SWCNTs, PEDOT: PSS, and ionic liquid possesses excellent conductivity and toughness.

The procedure for the preparation of the high-conductivity flexible electrode is described below:

1. Mix SWCNTs (10 mg) and Triton X-100 (50 mg) in 100 mL of deionized water, then disperse them by using a probe sonicator (600 W, 15min, on: 2s and off: 1s) in an ice-water bath to prevent overheating.

2. Add 5 wt% DMSO to the PEDOT: PSS solution and homogenize the mixture using a planetary mixer. Then, add 0.65 g of this modified PEDOT: PSS solution to the SWCNTs dispersion and further homogenize using a probe sonicator (600 W, 15min, on: 2s and off: 1s).

3. Centrifuge the resulting dispersion at 4000 rpm for 30 min and collect approximately 80% of the supernatant. Add ~ 10 mg of ionic liquid to the supernatant, followed by additional ultrasonic treatment (600 W, 8 min, 2 s on/1 s off) to ensure uniform mixing.

4. Perform vacuum filtration using a 0.22 µm PVDF membrane. Once filtration is complete and the film has dried, rinse it briefly with an IPA/acetone solution (v/v = 1:1) to remove residual Triton X-100. Subsequently, rinse the film thoroughly with deionized water.

5. The resulting film was clamped between two glass plates and dried in a vacuum oven (80 ℃, 4 h), and the flexible electrode could be easily peeled off after cooling to room temperature.

**Figure S12. (a) Schematic diagram of the ionic actuator fabrication procedure. Cross-sectional SEM image (b) and stress-strain curve (c) of the device.**

The oriented porous PVDF-HFP film is cut into the desired shape, and several drops of pristine electrolyte are applied on its surface. The ternary electrode mentioned above is then laminated onto the coated side, and the same procedure is repeated on the opposite surface. To ensure strong interfacial adhesion, the assembly is compressed using a glass slide and binder clip. The applied pressure was measured to be approximately 20 kPa, which provided sufficient contact between the electrode and electrolyte while preserving the aligned porous channels within the electrolyte (Figure S12b). A layer of non-stick release paper is inserted between the device and the slide to facilitate demolding. The assembled structure is heated at 60 °C for 2 hours and subsequently cooled to obtain the ionic actuator. The assembled device exhibits a Young’s modulus of approximately 58.8 MPa, providing sufficient mechanical stiffness for various micromanipulation and gripping applications.

**Figure S13. Comparative processability of Nafion and PVDF-HFP.**

The electrolytes commonly used in ionic actuators could be broadly categorized into two types: Nafion and PVDF-HFP. Nafion is well known as an amphiphilic block-like ionomer featuring nanophase separation, in which hydrophilic ionic clusters are embedded within a hydrophobic backbone. These ionic domains effectively facilitate ion transport; however, this structural characteristic also renders the ion transport behavior of Nafion highly sensitive to environmental conditions, particularly temperature and humidity. In contrast, PVDF-HFP is a hydrophobic polymer that does not intrinsically form well-defined ion transport channels. Nevertheless, its excellent processability and low cost make it an attractive candidate for practical applications. In this work, two different solvents, DMAc and 1,4-dioxane, are employed to process PVDF-HFP. Due to its low freezing point (~ -20 °C), DMAc is generally unsuitable for freeze-drying and is typically used for solution casting. 1,4-dioxane possesses a higher freezing point (~ 11.8 °C), which enables not only conventional casting but also freeze-drying to fabricate porous electrolyte structures. Therefore, this study systematically compares the morphological evolution of electrolytes prepared via different processing routes and evaluates their impact on actuator performance.

**Figure S14. Performance comparison between Nafion and PVDF-HFP.** a) A comparison of the chemical structures of Nafion and PVDF-HFP, along with their film formation behavior during the freeze-drying process. The right panel further displays the transparent films produced via solution casting from the respective polymer solutions. DSC data (b) and stress-strain curves (c) of Nafion and PVDF-HFP films. d) SEM of Dioxane-FD PVDF-HFP film with conventional DMAc-cast Nafion reveals that the former exhibits a distinct oriented porous structure. A systematic comparison among Dioxane-cast PVDF-HFP, DMAc-cast Nafion, and Dioxane-FD PVDF-HFP films is conducted in terms of ionic liquid absorption (e), ion mobility (f), and interfacial adhesion (g). Except for ion mobility, in which Nafion exhibits a marginal advantage, the Dioxane-FD PVDF-HFP films show marked superiority in other aspects. h) Overall performance comparison of DMAc-cast Nafion and Dioxane-FD PVDF-HFP.

Constructing oriented ion transport channels within electrolytes represents an effective strategy for enhancing the performance of ionic actuators. In this work, directional freeze-drying is employed to investigate two representative electrolyte materials: commercial Nafion aqueous solution and PVDF-HFP dissolved in 1,4-dioxane. The resulting Nafion films show severe cracking, while PVDF-HFP yielded continuous, porous membranes. This discrepancy could be attributed to differences in molecular structure and interactions, as shown in Figure S14a. Nafion, with four fluorine atoms per backbone unit, exhibits limited chain flexibility, and its sulfonic acid side groups possess a strong affinity for water, leading to non-uniform dehydration and localized stress accumulation, which induces film rupture. However, PVDF-HFP features a less fluorinated backbone with only two fluorine atoms, resulting in greater structural pliability. Solvent sublimation through the crystalline matrix during freeze-drying promotes uniform internal stress release, thereby preserving membrane structural integrity. The DSC results (Figure S14b) support this hypothesis, showing a glass transition temperature of approximately 88.27 °C for Nafion and 52.92 °C for PVDF-HFP, indicating the superior suitability of PVDF-HFP for freeze-drying. In comparison to those prepared by the casting method, the PVDF-HFP films exhibit a higher degree of opacity than Nafion films. This difference may stem from the high rigidity of PVDF-HFP fluorocarbon backbone, which promotes dense molecular packing and enhances light scattering. Conversely, the sulfonic acid groups in Nafion promote the formation of hydrophilic ionic channels within a predominantly amorphous matrix, resulting in a phase-separated but optically homogeneous structure that enhances optical homogeneity. These structural differences give rise to distinct mechanical behaviors, with PVDF-HFP films showing a substantially higher Young’s modulus (~ 282.2 MPa) and Nafion films demonstrating superior elongation (> 300%) due to inherent hydrophilicity (Figure S14c).

Given the inability of freeze-dried Nafion to form an intact porous structure, a systematic comparison is conducted among DMAc-cast Nafion, Dioxane-cast PVDF-HFP, and Dioxane-FD PVDF-HFP with respect to ionic liquid absorption, ion mobility, interfacial adhesion, and cost. Dioxane-FD PVDF-HFP film shows a well-aligned porous morphology, in contrast to the dense structure of casting films (Figure S14d), resulting in a markedly higher ionic liquid uptake of 163.4%, significantly exceeding that of DMAc-cast Nafion and Dioxane-cast PVDF-HFP film (Figure S14e). Ion mobility is further investigated by applying a voltage across the film and using the time required for the current to decay to 90% of its initial value as an indicator of ion mobility. Nafion possesses well-developed hydrophilic nanochannels formed by sulfonic groups, enabling efficient and continuous ion transport, which results in a high ion mobility of approximately 3.38×10^-5^ cm^2^ V^-1^ s^-1^. By comparison, Dioxane-cast PVDF-HFP film lacks such intrinsic ion pathways and primarily relies on ion diffusion under an electric potential, leading to a lower mobility of about 4.36×10^-6^ cm^2^ V^-1^ s^-1^. However, Dioxane-FD PVDF-HFP films achieve an enhanced ion mobility of 2.57×10^-5^ cm^2^ V^-1^ s^-1^, albeit slightly lower than that of Nafion, yet approximately seven times higher than the Dioxane-cast PVDF-HFP films. Higher mobility clearly demonstrates that the channels formed by freeze-drying effectively facilitate ion mobility (Figure S14f). Moreover, effective adhesion between the electrode and electrolyte is essential for enhancing ion transport efficiency and electrochemical stability in ionic actuators. The porous surface of Dioxane-FD PVDF-HFP provides abundant interfacial contact sites, resulting in the highest peel strength (Figure S14g). Most importantly, PVDF-HFP costs nearly one-tenth as much as Nafion, as shown in the radar chart (Figure S14h).

**Figure S15. Systematic comparison of ionic actuators based on different PVDF-HFP electrolytes in terms of displacement (a), response time (b), and strain variation under different temperatures (c-d).**

Figure S15a shows the displacement of actuators based on the different PVDF-HFP electrolytes. The actuator incorporating the Dioxane-FD PVDF-HFP demonstrates the highest displacement, which is nearly ten times greater than that of the actuator prepared by DMAc-cast film (Voltage: 2 V). Correspondingly, the response speed indicates that the freeze-dried sample achieves the fastest actuation response, reaching approximately 0.8 mm/s. Due to the hydrophobic nature of PVDF-HFP, the strain is relatively insensitive to temperature variations. The casting samples exhibit a modest performance decrease at elevated temperatures, likely due to ionic liquid leakage, whereas the Dioxane-FD PVDF-HFP remains stable, indicating enhanced ionic liquid retention enabled by oriented porous structure (Figure S15c-d). This further confirms that directional freeze-drying is well-suited for the fabrication of high-performance ionic actuators.

**E:** **Performance optimization of freeze-dried films**

**Figure S16. Schematic illustration of the freeze-drying (a) and the effects of temperature on morphology (b) and actuator performance (c-e).**

Directional freeze-drying is a common technique for constructing an oriented porous structure. As illustrated in Figure S16a, a mold with PTFE sidewalls and a copper base in direct contact with liquid nitrogen established a vertical temperature gradient, guiding the upward growth of solvent crystals. Subsequent sublimation of the frozen solvent preserves the framework, yielding vertically aligned channels. This process is sensitive to factors such as temperature, time, and solute concentration. Here, the effect of solution temperature (50~80  °C) on film morphology is investigated. Taking the film prepared at 80  °C as an example, the cross-sectional morphology of the resulting film could be divided into three distinct regions:

Region I (bottom layer): A dense structure resembling casting films, likely caused by the rapid evaporation of low-boiling-point 1,4-dioxane before effective freezing.

Region II (middle layer): A stable vertically aligned porous structure is established as underlying particles serve as nucleation sites, directing the anisotropic growth of solvent crystals and yielding the desired channel morphology.

Region III (top layer): A loose, dendritic, and poorly oriented structure, attributed to higher solvent content and smaller temperature gradients.

Figure S16b illustrates that with increasing solution temperature, the extents of Region I and Region III gradually diminish, likely due to reduced film formation at higher temperatures. Concurrently, Region II expands, and its structural alignment becomes more pronounced, which is critical for the performance of the actuator. Devices fabricated from films prepared at higher temperature gradients exhibit enhanced actuation, confirming that aligned porous channels significantly promote ion mobility (Figure S16c-e). Thus, a large vertical temperature gradient during freeze-drying is critical for optimizing film structure and actuator performance.

**Figure S17. Morphological variations of PVDF-HFP films under varying PVDF-HFP and ionic liquid concentrations (a). Changes in impedance, thickness, and ionic conductivity of freeze-dried film prepared by different PVDF-HFP concentrations (b-c). Performance analysis of devices fabricated from the above electrolyte (d-e).**

The morphological evolution of electrolytes prepared with varying concentrations of PVDF-HFP and ionic liquid is shown in Figure S17a. At a low PVDF-HFP concentration (7 wt%), the high solvent content leads to the formation of a lamellar structure with large pores after freeze-drying. As the PVDF-HFP concentration increases, this sheet-like structure transitions into an anisotropic porous channel. Further increasing the PVDF-HFP concentration results in a reduction of pore size and decreased structural alignment. Among these, the morphology obtained at 9 wt% PVDF-HFP exhibits the optimal structural anisotropy. Based on this ratio, we further investigated the effect of ionic liquid concentrations. Optical images reveal that excessive ionic liquid content induces tearing during the freeze-drying process, suggesting the disruption of the PVDF-HFP polymer network. SEM analysis confirms that when the ionic liquid ratio exceeds 2.0, the aligned porous channels collapse. Therefore, the ionic liquid ratio should be maintained below this threshold. Figures S17b-c present the variations in impedance, thickness, and ionic conductivity of electrolytes with different PVDF-HFP concentrations. Although the impedance remains relatively constant, increasing PVDF-HFP concentrations leads to thicker films, resulting in a gradual increase and eventual saturation in ionic conductivity. Figures S17d-e illustrate the corresponding performance of the fabricated ionic actuators. Devices based on the 9 wt% PVDF-HFP show the largest CV curve and highest actuation force, indicating that this formulation provides the optimal structure for ion mobility and is therefore selected for further investigation.

**Figure S18. Analysis of CV curve, impedance (a), strain response (b), and blocking force (c) of PVDF-HFP films prepared by DMAc-cast, Dioxane-cast, and Dioxane-FD under varying ionic liquid concentrations.**

Regardless of the processing method, the CV curve of the PVDF-HFP based actuators increases with the ionic liquid concentration, accompanied by a reduction in impedance. Notably, at all ionic liquid concentrations, the freeze-dried films consistently outperform the casting films, indicating a higher retention of ionic liquid and confirming that the freeze-drying process significantly enhances device performance. Figures S18b-c further present the corresponding strain and blocking force data. Across all film types, increasing the ionic liquid content leads to larger strain, but the strain gradually saturates. Meanwhile, the blocking force of the device begins to decline as it approaches saturation, which may be attributed to a reduction in the Young’s modulus caused by excessive ionic liquid.

**Figure S19. Analysis of morphology (a), CV curve (b), impedance (c), and strain response (d) of PVDF-HFP films prepared with different freezing durations.**

Figure S19a shows the morphological evolution of the films at different freezing durations. Freezing for 5 min results in the formation of uniform porous channels, and further increasing the time leads to enlarged pore sizes, which slightly degrades the device performance. Devices with a freezing time of approximately 10 min exhibit the best actuation performance.

**Figure S20. Electrolytes with different thicknesses are prepared by freeze-drying different volumes of precursor solutions, and the corresponding devices are evaluated through cyclic voltammetry (a), impedance (b), strain (c), and blocking force (d).**

Using a larger solution volume during freeze-drying results in thicker electrolytes, which impose greater resistance to ion mobility and typically lead to a reduction in strain output. However, the generated blocking force is significantly enhanced.

**F:** **Multifunctional applications of the actuator**

**Figure S21. (a-b) Schematic illustration of the ionic actuator gripper adapter and its integration with a robotic manipulator. (c) Grasping procedure of the gripper based on Dioxane-FD PVDF-HFP electrolyte.**

**Figure S22. Control circuit for the bio-inspired robotic fish.**

The battery is used to independently power the microcontroller (ATtiny2313A-MUR), voltage regulator (TPS74529PQWDRVRQ1), and H-bridge motor (DRV8837DSGT). To ensure stable operation of the voltage regulator, both input (C0805C105K3RACTU) and output capacitors (C0805C224K1RACAUTO, C0805C104K5RACTU) are added. The output voltage from the regulator is compared with the signal from the microcontroller to determine the forward and reverse functions of the motor, thereby generating an alternating signal used to drive the ionic actuator.

**Table S1. Comparison of solvent physical properties**

| **Property** | **DMAc** | **1,4-Dioxane** |
| --- | --- | --- |
| **Crystallization behavior**  **upon cooling** | Tends to vitrify | Readily crystallizes |
| **Melting point** | -20 ℃ | 11.8 ℃ |
| **Solvent polymer**  **interaction strength** | Strong | Moderate |
| **Dielectric constant** | ~37.8 | ~2.2 |
| **Vapor pressure (25 ℃)** | ~ 2 mmHg | ~ 38 mmHg |
| **Boiling point (℃)** | 165-166 | 101.1 |
| **Evaporation rate** | Slow | Fast |
| **Freeze-drying compatibility** | Not suitable | Suitable |

**Table S2. Performance comparison of robotic fish actuators**

| Actuator | Robot | Electric input (V) | Weight (g) | Actuator mass fraction (%) | Body length (cm) | Max. Velocity  (BL/s) | Ref. |
| --- | --- | --- | --- | --- | --- | --- | --- |
| FEDEA | Tethered swimming robot | 7500 | 25.1 | > 50 | 22 | 0.009 | ^[4]^ |
| DEA | Tethered swimming robot | 4800 | 14.3 | > 50 | 10 | 0.77 | ^[5]^ |
| DEA | Untethered snailfish robot | 7000 ~ 9000 | -- | -- | 22 | 0.45 | ^[6]^ |
| DEA | Untethered electro-ionic fish | 8000 ~ 10000 | 90.3 | -- | 9.3 | 0.69 | ^[7]^ |
| SMA | Tethered soft robotic fish | 50 | -- | -- | ~ 20 |  | ^[8]^ |
| SMA | Tethered stingray-inspired soft swimmer | 3.3 (A) | -- | -- | ~ 20 | 0.2 | ^[9]^ |
| SMA | Untethered soft robotic fish | 3.3 | ~ 100 | ~ 8 | ~ 10 | 0.38 | ^[10]^ |
| LMAM | Untethered bionic robotic fish | 3.7 | -- | -- | ~ 5 | 0.125 | ^[11]^ |
| IPMC | Tethered biomimetic robotic swimmer | 3 | 5.05 | -- | 8.75 | 0.097 | ^[12]^ |
| IPMC | Untethered robotic manta ray | 4 | 55 | ~ 25.5 | 11 | 0.067 | ^[13]^ |
| IPMC | Untethered robotic fish | 3.3 | 290 | ~ 5 | 20 | 0.104 | ^[14]^ |
| This work | **Untethered robotic fish** | **3.3** | **~ 17** | **~ 0.5** | **~ 9** | **~ 0.15** |  |

Principle: Fluid electrode dielectric elastomer actuator (FEDEA); Dielectric elastomer actuator (DEA); Shape memory alloy actuator (SMA); Liquid metal artificial muscle (LMAM); Ionic polymer metal composite (IPMC)

**References**

1	Zhu, Z., Chen, H., Chang, L. & Li, B. Dynamic model of ion and water transport in ionic polymer-metal composites. *AIP Advances* 4, no. 1 (2011): 040702.

2	Nemat-Nasser, S. Micromechanics of actuation of ionic polymer-metal composites. *Journal of Applied Physics* 5, no. 92 (2002): 2899-2915.

3	Tjaden, B., Cooper, S. J., Brett, D. J. L., Kramer, D. & Shearing, P. R. On the origin and application of the Bruggeman correlation for analysing transport phenomena in electrochemical systems. *Current Opinion in Chemical Engineering*, no. 12 (2016): 44-51.

4	Christianson, C., Goldberg, N. N., Deheyn, D. D., Cai, S. & Tolley, M. T. Translucent soft robots driven by frameless fluid electrode dielectric elastomer actuators. *Science Robotics* 17, no. 3 (2018): eaat1893.

5	Wang, S., Huang, B., McCoul, D. *et al.* A soft breaststroke-inspired swimming robot actuated by dielectric elastomers. *Smart Materials and Structures* 4, no. 28 (2019): 045006.

6	Li, G., Chen, X., Zhou, F. *et al.* Self-powered soft robot in the Mariana Trench. *Nature* 7848, no. 591 (2021): 66-71.

7	Li, T., Li, G., Liang, Y. *et al.* Fast-moving soft electronic fish. *Science Advances* 4, no. 3 (2017): e1602045.

8	Bartlett, M. D., Kazem, N., Powell-Palm, M. J. *et al.* High thermal conductivity in soft elastomers with elongated liquid metal inclusions. *Proceedings of the National Academy of Sciences* 9, no. 114 (2017): 2143-2148.

9	Ohm, Y., Pan, C., Ford, M. J. *et al.* An electrically conductive silver–polyacrylamide–alginate hydrogel composite for soft electronics. *Nature Electronics* 3, no. 4 (2021): 185-192.

10	Clark, A. J., Tan, X. & McKinley, P. K. Evolutionary multiobjective design of a flexible caudal fin for robotic fish. *Bioinspiration & Biomimetics* 6, no. 10 (2015): 065006.

11	Shu, J., Ge, D.-A., Wang, E. *et al.* A Liquid Metal Artificial Muscle. *Advanced Materials* 43, no. 33 (2021): 2103062.

12	Shen, Q., Wang, T., Liang, J. & Wen, L. Hydrodynamic performance of a biomimetic robotic swimmer actuated by ionic polymer–metal composite. *Smart Materials and Structures* 7, no. 22 (2013): 075035.

13	Chen, Z., Um, T. I. & Bart-Smith, H. Bio-inspired robotic manta ray powered by ionic polymer–metal composite artificial muscles. *International Journal of Smart and Nano Materials* 4, no. 3 (2012): 296-308.

14	Chen, Z., Shatara, S. & Tan, X. Modeling of Biomimetic Robotic Fish Propelled by An Ionic Polymer–Metal Composite Caudal Fin. *IEEE/ASME Transactions on Mechatronics* 3, no. 15 (2010): 448-459.
